# Differential affinity chromatography reveals a link between *Porphyromonas gingivalis*–induced changes in vascular smooth muscle cell differentiation and the type 9 secretion system

**DOI:** 10.3389/fcimb.2022.983247

**Published:** 2022-11-22

**Authors:** Priscilla L. Phillips, Xiao-jun Wu, Leticia Reyes

**Affiliations:** ^1^ Microbiology and Immunology, A.T. Still University of Health Sciences, Kirksville College of Osteopathic Medicine, Kirksville, MO, United States; ^2^ Department of Pathobiological Sciences, University of Wisconsin - Madison, School of Veterinary Medicine, Madison, WI, United States

**Keywords:** *Porphyromonas gingivalis*, type 9 secretion system, far-western blot, affinity chromatography, *P. gingivalis*/aortic smooth muscle cell interactions

## Abstract

*Porphyromonas gingivalis* is implicated in adverse pregnancy outcome. We previously demonstrated that intrauterine infection with various strains of *P. gingivalis* impairs the physiologic remodeling of the uterine spiral arteries (IRSA) during pregnancy, which underlies the major obstetrical syndromes. Women diagnosed with IRSA also have a greater risk for premature cardiovascular disease in later life. The dysregulated plasticity of vascular smooth muscle cells (VSMCs) is present in both IRSA and premature cardiovascular events. We hypothesized that VSMCs could serve as a bait to identify *P. gingivalis* proteins associated with dysregulated VSMC plasticity as seen in IRSA. We first confirmed that dams with *P. gingivalis* A7UF-induced IRSA also show perturbed aortic smooth muscle cell (AoSMC) plasticity along with the *P. gingivalis* colonization of the tissue. The *in vitro* infection of AoSMCs with IRSA-inducing strain A7UF also perturbed AoSMC plasticity that did not occur with infection by non-IRSA-inducing strain W83. Far-Western blotting with strain W83 and strain A7UF showed a differential binding pattern to the rat aorta and primary rat AoSMCs. The affinity chromatography/pull-down assay combined with mass spectrometry was used to identify *P. gingivalis*/AoSMC protein interactions specific to IRSA. Membrane proteins with a high binding affinity to AoSMCs were identified in the A7UF pull-down but not in the W83 pull-down, most of which were the outer membrane components of the Type 9 secretion system (T9SS) and T9SS cargo proteins. Additional T9SS cargo proteins were detected in greater abundance in the A7UF pull-down eluate compared to W83. None of the proteins enriched in the W83 eluate were T9SS components nor T9SS cargo proteins despite their presence in the prey preparations used in the pull-down assay. In summary, differential affinity chromatography established that the components of IRSA-inducing *P. gingivalis* T9SS as well as its cargo directly interact with AoSMCs, which may play a role in the infection-induced dysregulation of VSMC plasticity. The possibility that the T9SS is involved in the microbial manipulation of host cell events important for cell differentiation and tissue remodeling would constitute a new virulence function for this system.

## Introduction

*Porphyromonas gingivalis* is implicated in a variety of obstetric complications including early pregnancy loss, early-onset preeclampsia with and without fetal growth restriction, preterm labor, and the preterm premature rupture of membranes (Barak et al., 2007; León et al., 2007; Swati et al., 2012; Chaparro et al., 2013; Ibrahim et al., 2015; Vanterpool et al., 2016). Using a rat model of chronic periodontitis, we previously demonstrated that intrauterine colonization by various strains of *P. gingivalis* impairs the physiologic remodeling of the uterine spiral arteries (IRSA) during pregnancy (Phillips et al., 2018; Tavarna et al., 2020; Tavarna et al., 2022). Since IRSA underlies the major obstetrical syndromes, this evidence provided a common mechanistic link whereby *P. gingivalis* could promote such a diverse array of pregnancy complications. However, the microbial virulence factors involved in this disorder remain unknown.

Women with a history of IRSA are also at greater risk for having a premature coronary event such as coronary arterial disease and restenosis after the affected pregnancy (Veerbeek et al., 2016; Brosens et al., 2017; Benagiano et al., 2021). A common link between these cardiovascular disorders and IRSA is the dysregulation of vascular smooth muscle cell (VSMC) plasticity (Whitley and Cartwright, 2010; Brosens et al., 2011; Low et al., 2019; Nankivell et al., 2020). In the systemic vasculature, inappropriate VSMC responses to transforming growth factor beta (TGF-β) signaling are implicated in both coronary arterial disease and restenosis (Suwanabol et al., 2011; Low et al., 2019). Within the placental bed, the physiologic dedifferentiation of spiral arterial VSMCs is driven by transforming growth factor beta (TGF-β) signaling (Liu et al., 2019). It is unknown if perturbed TGF-β signaling plays a role in dysfunctional spiral arterial VSMC plasticity seen in IRSA, but there is an association between IRSA and perturbed signaling by members of the TGF-β superfamily in our rat model (Phillips et al., 2018).

Several reports show that the *in vitro* infection of aortic smooth muscle cells (AoSMCs) with *P. gingivalis* alters aortic VSMC phenotype switching to potentially pro-atherogenic responses (Zhang et al., 2013; Zhang et al., 2016; Park et al., 2020). Infection with *P. gingivalis* also enhances aortic intimal hyperplasia with experimental aortic injury (Hokamura et al., 2010), showing that *P. gingivalis* perturbs VSMC homeostatic responses *in vivo*. Moreover, *P. gingivalis* outer membrane vesicles and gingipains affect VSMC phenotype switching *in vitro* (Cao et al., 2015; Yang et al., 2016; Zhang et al., 2016). Among these studies is evidence that the perturbation of the TGF-β signaling axis plays a role in *P. gingivalis*-mediated dysfunction in VSMCs (Zhang et al., 2013; Yang et al., 2016; Zhang et al., 2016). Since host cell signaling events depend on receptor activation, we proposed that VSMCs could serve as a bait to identify *P. gingivalis* proteins that interact with the host cell and may be associated with abnormal VSMC phenotypes.

We first established the feasibility of using rat AoSMCs as a surrogate for spiral arterial smooth muscle that cannot be isolated as a purified cell population from the smooth muscle–rich uterus. To identify VSMC/*P. gingivalis* interactions specific to IRSA, we compared non-IRSA-inducing *P. gingivalis* strain W83 (Phillips et al., 2018) to IRSA-inducing *P. gingivalis* strain A7UF (Tavarna et al., 2020). Far-Western blotting demonstrated that IRSA-inducing A7UF proteins bound differently to the rat aorta and AoSMCs compared to nonIRSA-inducing W83. We then used affinity chromatography (pull-down assay) combined with mass spectrometry to identify *P. gingivalis*/AoSMC protein interactions specific to IRSA. We found a strong interaction between AoSMCs and components of the IRSA-inducing A7UF type 9 secretion system (T9SS) that was not evident with non-IRSA-inducing W83. Moreover, A7UF T9SS cargo proteins that are putative virulence factors such as CPG70, RgpB gingipains, and peptidylarginine deiminase showed enhanced binding to AoSMCs compared to W83. Overall, our results suggest that the *P. gingivalis* T9SS may play a role in VSMC dysfunction, perhaps by facilitating the entry of various microbial virulence factors into host cells.

## Materials and methods

### Rat tissue collection and processing

The rat aorta and primary AoSMCs used in this study were obtained from Sprague–Dawley (SD) rats (RRID : RGD_734476) used in a previous study (Phillips et al., 2018). All experimental protocols were approved by the University of Wisconsin Institutional Animal Care and Use Committee (protocol #V005576). After euthanasia, both the thoracic and abdominal portions of the aorta from uninfected control animals was isolated and rinsed in sterile phosphate-buffered saline (PBS). The adventitia was removed aseptically, flash-frozen in liquid nitrogen, and stored at 80°C until protein extraction.

AoSMCs were isolated by the double-collagenase method (Villa-Bellosta and Hamczyk, 2015). The entire thoracic and abdominal aorta was aseptically removed. Collagenase type 2 (Worthington Biochemical Corp. catalog # LS004174) was used for tissue dissociation. AoSMCs were cultured in Dulbecco's Modified Eagle Medium/Nutrient Mixture F-12 (DMEM/F-12) (catalog # 12-719F, Lonza, Walkersville, MD, USA) with 10% fetal calf serum (FBS) (catalog #FB-12, Omega Scientific) and gentamicin sulfate with amphotericin-B (catalog #CC-4083, Lonza, Walkersville, MD, USA). AoSMC purity was ≥90% verified by positive staining for α-smooth muscle actin (RRID: AB_2223019). For all experiments, AoSMCs were used at passage 7.

### *P. gingivalis* culture and protein extraction

*P. gingivalis* W83 and A7UF were provided by Dr. Ann Progulske-Fox (University of Florida, USA). All experiments, including previous *in vivo* infection studies (Phillips et al., 2018; Tavarna et al., 2020) were performed using the same bacterial working stocks. For these studies, both strains were cultured at the same time under the same conditions on tryptic soy agar supplemented with hemin (5 mg/L), yeast extract (5 g/L), L-cysteine (0.5 g/L), and vitamin K3 (1 mg/L), passaged once. Bacteria for *in vitro* infection and for proteomics were prepared from cultures that were 48 h old. Bacterial colonies from agar were chosen to approximate bacterial conditions in tissue biofilms that we have observed in placental bed specimens from infected animals (Tavarna et al., 2020; Tavarna et al., 2022).

For *in vitro* infection experiments, bacterial colonies were suspended in sterile PBS and their concentration was estimated by optical density readings taken at 550 nm. Inoculates were then prepared by dilution in sterile DMEM/F-12 media. Bacterial concentrations in inoculates were confirmed by culture as previously described (Tavarna et al., 2020).

For proteome and affinity chromatography experiments, bacterial colonies were suspended in sterile PBS with protease inhibitors (Halt™ Protease Inhibitor Cocktail, catalog #87785, Thermo Fisher Scientific), pelleted by centrifugation (3,000 x g for 5 min) and processed immediately. Bacterial proteins were extracted with B-PER™ Bacterial Protein Extraction Reagent (catalog #90079, Thermo Fisher Scientific) with a Halt™ protease inhibitor cocktail and a Lysonase Bioprocessing Reagent (catalog #71230, Millipore Sigma) according to the manufacturer’s instructions. Bacterial protein extracts were dialyzed overnight (6–8 MW cut-off) in a 0.1 M sodium phosphate buffer, 0.1 M NaCl pH 7.4 at 4°C before use. The detergent-free preparations of total soluble bacterial protein were used to cast the widest net for the *de novo* capture of on-column protein–protein interactions and facilitate the subsequent LC/MS/MS identification of eluted *P. gingivalis* proteins.

For the far-Western blot, bacterial protein extracts were labeled with EZ-link™-NHS-SS-PEG4-Biotin (catalog #21442, Thermo Fisher Scientific). During biotin labeling, bacterial proteins were incubated on ice for 2 h on a rotating shaker and then dialyzed overnight with Spectra/Por^®^ 1 Dialysis Membranes, MWCO 6000 to 8000 (Spectrum laboratories) in a 0.1 M sodium phosphate buffer, 0.1M NaCl pH 7.4 at 4°C. The biotin labeling of both *P. gingivalis* strains was confirmed by Western blot.

### Gene expression analysis

Total RNA from aortic tissue and AoSMCs was extracted with a TRIzol™ Reagent (Life Technologies, Cat# 15596-018) according to the manufacturer’s instructions. RNA quality and quantity were assessed by capillary electrophoresis (Agilent RNA 6000, Agilent Technologies, Inc., Santa Clara, CA, USA). A select panel of genes was evaluated using the following Quantitect primers from Qiagen Inc. (Germantown, MD, USA): β-actin, *Actb* as the reference gene (catalog# QT00193473), interleukin-1β, *Il1b* (Qiagen catalog# QT00181657), the tumor necrosis factor, *Tnf* (Qiagen catalog# QT00178717), chemokine monocyte chemotactic protein (*Mcp1/Ccl2*) [forward:5’-ATGAGTCGGCTGGAGAACTA-3’, reverse: 5’ACTTCTGGACCCATTCCTTATTG-3’], *Tgfb1* (Qiagen catalog# QT00187796), endoglin, *Eng* [forward: 5’-GCGTCCTTGTTCCAAACATTC-3’, reverse 5’-ACAGCAAGAAGAGGCAAGAG-3’], e-Selectin (*Sele*) [forward:5’-GTATCCATCCATCCCACAGAAG-3’, reverse: 5’-CAGTTGTGTCCACTCGATAGTT-3’], Icam-1, *Icam1* [forward: 5’-GTATCCATCCATCCCACAGAA-3’, reverse: 5’-CAGTTGTGTCCACTCGATAGTT-3’, matrix metalloproteinase 2, *Mmp2* [forward: 5’-TGGACTGGAGAAGGACAA-3’, reverse: 5’-CTGCTGTATTCCCGACCATTA-3’], matrix metalloproteinase 9, *Mmp9* [forward: 5’-CCCAACCTTTACCAGCTACTC-3’, reverse: 5’-GTCAGAACCGACCCTACAAAG-3”], and the tissue inhibitor of metalloproteinase-1, Timp1[forward:5’-TGGCATCCTCTTGTTGCTATC-3’, reverse: 5’-CCTTATAACCAGGTCCGAGTTG-3’]. Real-time qPCR was performed with the Light Cycler^®^ 96 real-time PCR System (Roche Applied Science, Indianapolis, IN, USA) as previously described (Tavarna et al., 2022).

Gene expression data were either analyzed by Student’s t-test for two groups or by one-way analysis of variance (ANOVA) for three groups followed by the Tukey multiple comparison test if ANOVA indicated a significant difference among group means. Raw data measurements were transformed prior to ANOVA testing if the Bartlett test showed that variances were significantly different among groups. Testing was performed with Prism 9.4.0 Software (GraphPad). For all testing, P < 0.05 was considered significantly different.

### Immunofluorescent staining of rat aortic tissue

Aortic sections from sham-inoculated controls and *P. gingivalis* A7UF- infected dams were performed as previously described [10,11]. For antigen retrieval, specimens were incubated with a citrate buffer pH 6 [10 mM sodium citrate with 0.05% Tween 20] at 95°C for 5–10 min. The primary antibodies used in this study were mouse anti-smooth muscle actin (Abcam Cat# ab76549, RRID: AB_2223019) to detect VSMCs, rabbit anti-TGF-β1 (Abcam Cat# ab25121, RRID: AB_2271652), and rabbit-anti endoglin (MyBiosource.com cat#MBS2001783, RRID: AB_29223167). All antibodies were used at a dilution of 1:200. *P. gingivalis* was detected with a rabbit polyclonal antibody used at a dilution of 1:1,000, which was previously validated for specificity and its ability to detect multiple *P. gingivalis* strains (Vanterpool et al., 2016; Phillips et al., 2018). Mouse IgG isotype control (Thermo Fisher Scientific Cat# 31903) and normal rabbit serum (Thermo Fisher Scientific Cat# 31883) were used at a dilution of 1:200. The secondary antibodies used for detection were goat anti-rabbit ALEXA 647 (catalog # A-21244) and goat anti-mouse ALEXA 594 (catalog # A-11005) (Thermo Fisher Scientific, Waltham, MA, USA); both were used at a concentration of 2 µg/ml. Stained tissue sections were visualized and imaged using an EVOS AutoFL microscope system (Life Technologies, Grand Island, NY, USA). Camera settings for the imaging of fluorescent stains were optimized using the tissue section with the highest positive fluorescent signal. Once optimized, the settings were then kept the same while imaging all other tissue sections for a specific staining experiment.

### Aortic smooth muscle cell migration and proliferation assays

Migration assays were performed as previously described (Brown et al., 1994) with the following modifications. First, AoSMCs were serum-depleted for 24 h in 1% fetal calf serum (FBS) in DMEM/F12 media) before inoculation with a sterile medium or *P. gingivalis* A7UF or W83 at a multiplicity of infection (MOI) = 10 in 1% FBS in DMEM/F12 media. The MOI of each inoculate was confirmed by culture as previously described (Tavarna et al., 2020). Cells were maintained under serum depletion for another 24 h before they were transferred to 8 mm Transwell inserts (Falcon^®^ Cell Culture Inserts by Corning, catalog # 353097). Briefly, adherent cells from all treatment groups were removed by trypsin treatment, washed once with sterile PBS, counted, pelleted by centrifugation (800 x g), and resuspended in 5% FBS in DMEM/F12 to a cell density of 1 × 10^5^ cells/ml (10^4^ cells/well). Bottom wells received 5% FBS in DMEM/F-12 media (negative) or vitronectin (3 µg/ml, catalog # 5051, Advanced Biomatrix.com) in DMEM/F-12 + 5% FBS and incubated at 37°C and 5% CO_2_. After 6 h, the remaining cells within the Transwell insert were removed, the cells attached to the bottom membrane were fixed and stained (Diff-Quik Stain Set, Siemens Medical System). The calibrated images of the entire membrane were captured with an EVOS AutoFL imaging system, and cell numbers were normalized by the surface area (mm^2^).

For proliferation experiments, AoSMCs were seeded unto chamber slides (NUNC™ Lab-Tek™ II Chamber Slide system) (Thermo Fisher Scientific, catalog # 154534). After 24 h, cells were maintained for 24 h in 1% FBS in DMEM/F12 before inoculation with a sterile medium (control) or 10 MOI of A7UF or W83 suspended in 1% FBS in DMEM/F12. AoSMCs were incubated at 37°C in 5% CO_2_ for an additional 72 h with daily media changes (1% FBS in DMEM/F12). At the end of the incubation period, cells were fixed with 4% buffered formalin for 10 min, washed with PBS, permeabilized with 0.5% Triton-X for 10 min, and incubated with a blocking solution (2% goat serum, 1% bovine serum albumin, 0.1% Triton X-100, 0.05% Tween 20, and 0.05% sodium azide in 0.01 M PBS) for 30 min at room temperature. Proliferating cells were detected with a rabbit anti-proliferating cell nuclear antigen (PCNA) antibody (Thermo Fisher Scientific, catalog # PA5-27214, RRID: AB_2544690) diluted 1:200 in a blocking buffer. Isotype controls were stained with normal rabbit serum (Thermo Fisher Scientific Cat# 31883). Goat anti-rabbit ALEXA 647 (catalog # A-21244) was used for detection, and nuclei were counterstained with 4',6-diamidino-2-phenylindole (DAPI) (Thermo Fisher Scientific, ProLong™ Gold Antifade Mountant with DAPI). For each biological replicate, 10 calibrated images that spanned the entire well area were captured with an EVOS Auto FL imaging system. The percentage of proliferating cells was determined by dividing the number of cells in which the entire nucleus was PCNA positive by the total number of nuclei per well.

### Far-western blot

Proteins from the whole rat aorta were extracted with a T-PER™ Tissue Protein Extraction Reagent (catalog # 78510, Thermo Fisher Scientific) containing a Halt™ protease inhibitor cocktail (catalog #87785, Thermo Fisher Scientific). Membrane proteins were extracted from AoSMCs with the Subcellular Protein Fractionation Kit for Cultured Cells (catalog #78840, Thermo Fisher Scientific).

Equal amounts of protein from the rat aorta and AoSMCs were loaded in duplicate onto a 12.5% SDS gel (**Supplementary Figure S1** for gel layout). After electrophoresis, separated proteins were transferred to an Immobilon^®^-FL PVDF membrane (catalog # IPFL07810, Millipore-Sigma). The membrane was incubated in a blocking buffer [3% BSA/1% goat serum in TBS buffer] overnight at 4°C. The membrane was then cut in half so that one-half was incubated overnight at 4°C with biotin labeled *P. gingivalis* A7UF while the other was incubated with biotin labeled *P. gingivalis* W83, each at a concentration of 130 µg in 5 ml of a blocking buffer. The next morning, both membranes were washed three times in TBS-T before a 30-min incubation with IRDYE^®^ 800CW streptavidin (catalog # 926-32230, LI-COR) at a concentration of 1µl/5 ml of a blocking buffer. After incubation, membranes were washed three times, 15 min per wash with TBS-T. Both A7UF and W83 labeled membranes were imaged at the same time with an AzureC600 imaging system (Azure Biosystems, CA, USA).

### Detection of *P. gingivalis*/aortic smooth muscle cell protein interaction by pull-down assay

AoSMC surface proteins were used as “bait” to capture, detect, and compare total soluble *P. gingivalis* “prey” proteins from IRSA-inducing strain A7UF and nonIRSA-inducing strain W83. To prepare the bait, intact AoSMCs were first labeled with EZ-link™-NHS-SS-PEG4-Biotin (catalog #21442, Thermo Fisher Scientific) using the Pierce™ Cell Surface Protein Biotinylation and Isolation kit (catalog #A44390, Thermo Fisher Scientific) according to the manufacturer’s instructions. After biotin labeling, AoSMC membrane proteins were extracted with the Subcellular Protein Fractionation Kit for Cultured Cells (catalog #78840, Thermo Fisher Scientific) and dialyzed overnight with PBS before use. AoSMC protein extract quality and biotin labeling were checked by Western blot analysis (**Supplement File 1**, **Figure S2**). *P. gingivalis*/AoSMC protein interactions were determined with the Pierce™ Biotinylated Protein Interaction Pull-Down Kit (catalog # 21115, Thermo Fisher Scientific) as per the manufacturer’s instructions. The detergent-free preparations of total soluble bacterial proteins were used to cast the widest net for the *de novo* capture of on-column, strong protein–protein interactions and facilitate the subsequent LC/MS/MS identification of eluted *P. gingivalis* proteins. The optimization of pull-down assay conditions was confirmed by the silver staining of agarose gels that contained the samples of the serial eluates of bait control and bacterial samples (**Supplement File 1**, **Figure S3**). A ratio of one part AoSMCs (bait) to two parts *P. gingivalis* total soluble protein lysate (prey) was found to produce the greatest number of protein bands in the eluates. Briefly, immobilized streptavidin columns were loaded with the same concentration of the biotin-labeled AoSMC lysate (bait) while the negative (no bait) control columns received an equal volume of buffer. After incubation, streptavidin-available sites were blocked with a biotin-blocking solution. Then, an equal concentration of *P. gingivalis* A7UF or W83 total (prey) protein extracts, prepared as described above, were added to the AoSMC (bait)-loaded columns. The negative-bait control column received the same concentration of *P. gingivalis* A7UF or W83 (to detect non-specific binding), and the bait-only control column was loaded with the same volume of diluent buffer. All affinity columns were then incubated at 4°C for 48 h before washing (pH 5 buffer) and eluting with acidic buffer (pH 2.8). An aliquot of each eluate was screened for the presence of protein by gel electrophoresis and silver staining before samples were analyzed by NanoLc/MS/MS. The protein concentrations of each eluate was determined with a Nanodrop 1000 spectrophotometer (Thermo Fisher Scientific). The two series-matched eluate fractions that contained the most protein per group were selected and pooled for NanoLC/MS/MS analysis.

### *P. gingivalis* proteome analysis

Both *P. gingivalis* strains A7UF and W83 were cultured at the same time, and total soluble protein was extracted as described above. Two hundred micrograms of protein extracts from each strain were processed by “in liquid” digestion and bacterial proteins were identified by nano scale liquid chromatography-coupled to tandem mass spectrometry (NanoLC-MS/MS) as described below.

### Enzymatic “in liquid” digestion

*P. gingivalis*–soluble protein extracts for proteome analysis or captured protein eluates from the pull-down assay underwent TCA/acetone incubation for 30 min on ice to precipitate proteins (10% TCA in 50% acetone final vol:vol). The samples were spun for 10 min at room temperature at 16,000 x g, and pellets were washed twice with cold acetone. Generated protein extracts were resolubilized and denatured in 10 μl of 8 M urea in 50 mM NH_4_HCO_3_ (pH 8.5) and then subsequently diluted to 30 μl for the reduction step with 1.25 μl of 25 mM 1,4-Dithiothreitol (DTT) and 21.25 μl of 25 mM NH_4_HCO_3_ (pH 8.5). The samples were incubated at 56°C for 15 min and cooled on ice to room temperature, and then, 1.5 μl of 55 mM CAA (chloroacetaminde) was added for alkylation and incubated in darkness at room temperature for 15 min. The reactions were quenched by adding 4 μl of 25 mM DTT. Finally, 1.2 μl of a trypsin/LysC solution [100 ng/μl 1:1 trypsin (Promega) and LysC (FujiFilm) mix in 25 mM NH_4_HCO_3_] and 13.3 μl of 25 mM NH_4_HCO_3_ (pH8.5) was added to the 50 µl final volume. Digestion was conducted for 2 h at 42°C, and then, an additional 0.6 µl of the trypsin/LysC mix was added and digestion proceeded overnight at 37°C. The reactions were terminated by acidification with 2.5% TFA [trifluoroacetic acid] to a 0.3% final concentration.

### NanoLC-MS/MS

*P. gingivalis* protein “in liquid” digests were desalted using Agilent Bond Elut OMIX C18 solid phase extraction (SPE) pipette tips per the manufacturer’s protocol, and the peptides were eluted in 10 µl of 60%/40%/0.1% ACN/H_2_O/TFA. Dried to completion in the speed-vac and finally reconstituted in 12 µl of 0.1% formic acid, the peptides were analyzed by nanoLC-MS/MS using the Agilent 1100 nanoflow system (Agilent) connected to a hybrid linear ion trap-orbitrap mass spectrometer (LTQ-Orbitrap Elite™, Thermo Fisher Scientific) equipped with an EASY-Spray™ electrospray source (held at constant 35°C). The chromatography of peptides prior to mass spectral analysis was accomplished using a capillary emitter column (PepMap^®^ C18, 3 µM, 100 Å, 150 × 0.075 mm, Thermo Fisher Scientific) onto which 3 µl of extracted peptides were automatically loaded. The NanoHPLC system delivered solvents A: 0.1% (v/v) formic acid and B: 99.9% (v/v) acetonitrile, 0.1% (v/v) formic acid at 0.50 µl/min to load the peptides (over a 30-min period) and 0.3 µl/min to elute peptides directly into the nanoelectrospray with a gradual gradient from 0% (v/v) B to 30% (v/v) B over 80 min and concluded with a 5-min fast gradient from 30% (v/v) B to 50% (v/v) B, at which time a 5-min flash-out from 50%–95% (v/v) B took place. As peptides eluted from the HPLC-column/electrospray source survey MS scans were acquired in the Orbitrap with a resolution of 120,000 followed by the CID-type MS/MS fragmentation of 30 most intense peptides detected in the MS1 scan from 350 to 1,800 m/z; redundancy was limited by dynamic exclusion.

### Data analysis of LC/MS/MS data

Elite acquired MS/MS data files were converted to the mgf file format using MSConvert (ProteoWizard: Open Source Software for Rapid Proteomics Tools Development). The resulting mgf files were used to search against concatenated Uniprot *Rattus norvegicus* and *P. gingivalis* proteome databases (UP000002494 and UP00000588, respectively, 04/05/2021 download, 33,543 total entries) along with a cRAP common lab contaminant database (116 total entries) using in-house Mascot search engine 2.7.0 [Matrix Science] with fixed cysteine carbamidomethylation and variable methionine oxidation plus asparagine or glutamine deamidation. Peptide mass tolerance was set at 10 ppm and fragment mass at 0.6 Da. Protein identification, the significance of identification, and spectral-based quantification were done with Scaffold software (version 4.11.0, Proteome Software Inc., Portland, OR, USA). Peptide identifications were accepted if they could be established at greater than 88.0% probability to achieve an false discovery rate (FDR) less than 1.0% by the Scaffold Local FDR algorithm. Protein identifications were accepted if they could be established at greater than 8.0% probability to achieve an FDR less than 1.0% and contained at least two identified peptides. Protein probabilities were assigned by the Protein Prophet algorithm (Nesvizhskii et al., 2003). Proteins that contained similar peptides and could not be differentiated based on MS/MS analysis alone were grouped to satisfy the principles of parsimony. Proteins sharing significant peptide evidence were grouped into clusters.

The functional analysis of identified *P. gingivalis* proteins was expanded using Uniprot, InterPro, and EggNOG databases and tools. Proteins were initially sorted by clusters of orthologous gene (COG) classifications and keyed by identified functional domains and a putative subcellular location. Select Pfam functional domains in *P. gingivalis* W83 (pgi) were cross-referenced using the Kyoto Encyclopedia of Genes and Genomes (KEGG) database (e.g., Por_Secre_tail/PF18962) for record completeness. The analyses of each identified protein were cross-referenced against *P. gingivalis 33277* in all databases because of its broad use as the *P. gingivalis* reference strain. The corresponding W83 and 33277 genes of each identified protein were assessed for associated genes and functional pathways using STRING and cross-referenced to PubMed-indexed reports to further expand on annotations with experimentally supported functional data to facilitate classifications and data sorting. Identified and annotated proteins were grouped into one or more functional classification groups and sorted by total and relative expression (spectral count) in W83 and A7UF.

## Results

### IRSA-affected dams have evidence of aortic smooth muscle injury

**Figure 1:**
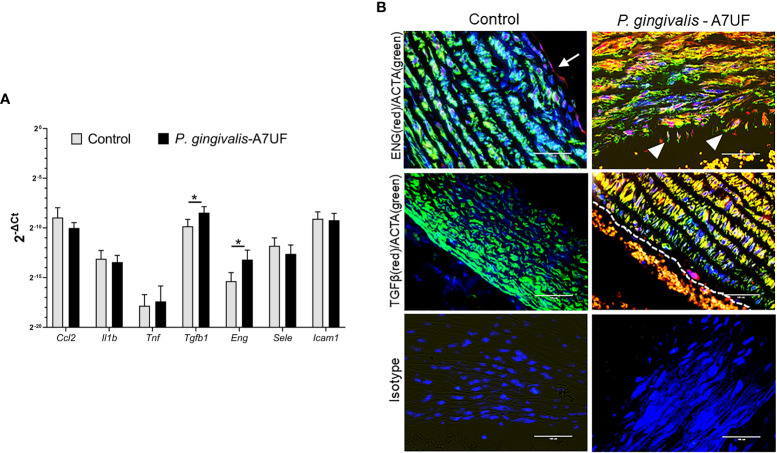
The aorta of IRSA affected dams has enhanced expression of TGF-β1 and endoglin signaling. **(A)** The descending aorta from pregnant sham (controls) and dams infected with IRSA-inducing *P. gingivalis* A7UF (n = 6) was examined for the expression of *Ccl2*, *Il1b*, *Tnf*, *Sele* (e-selectin), *Icam1*, *Tgfb1*, and *Eng* (endoglin). Gene expression was determined by quantitative real-time PCR (qRT-PCR) using β-actin as the reference gene. Values are the mean ± SD of 2^-ΔCt^ (n=5). *Indicates values statistically different by Student’s t-test. **(B)** *In situ* detection of TGF-β1(red) and Eng (red) protein in rat aortic arch sections from sham controls and dams infected with *P. gingivalis* A7UF. Aortic smooth muscle was identified by positive staining for α-actin (ACTA, green). Nuclei were stained with DAPI. Scale bar = 50 µm. White arrows point to Eng-positive endothelium. White arrowheads point to Eng-positive vascular smooth muscle cells (VSMCs) migrating into aortic intima. Dashed line demarcates the endothelial edge from the arterial lumen that contains autofluorescent red blood cells.

Presently, there are no methods that can isolate the enriched or purified populations of spiral arterial VSMCs because there are no established biomarkers that can distinguish spiral arterial VSMCs from the dominant population of uterine smooth muscle cells. Therefore, we sought to determine if AoSMCs could serve as a biologically relevant surrogate for spiral arterial VSMCs since women diagnosed with IRSA exhibit systemic arterial dysfunction during pregnancy that persists after giving birth (Hermes et al., 2013; Veerbeek et al., 2015; Harris et al., 2019). Moreover, our rat model of *P. gingivalis*–induced IRSA is created by a chronic oral infection of 3 months duration before breeding (Tavarna et al., 2020; Tavarna et al., 2022), which could promote systemic maternal vascular injury. The aorta from sham-inoculated controls and *P. gingivalis* A7UF-induced IRSA-affected dams were evaluated for vascular injury. The thoracic and abdominal aorta were first analyzed for the expression of genes involved in inflammation (MCP-1/Ccl2, interleukin-1β, and TNF), endothelial injury/activation (e-selectin and Icam-1), and remodeling (TGF-β1 and endoglin) (Tang et al., 2011; Tian et al., 2017). There was no difference in the expression of inflammatory cytokines (Ccl2, Il1b, and Tnf) nor endothelial injury (e-selectin and Icam-1) between the groups. However, both endoglin and TGF-β1 expression were increased in IRSA-affected animals (**Figure 1A**).

Aortic arch and valve specimens from sham-inoculated controls and IRSA-affected dams infected with *P. gingivalis* A7UF were immunostained for endoglin and TGF-β1. Both control and IRSA-affected dams showed endoglin staining in the aortic endothelium, but only IRSA-affected dams showed endoglin-positive VSMCs, some of which were seen migrating into the aortic intima (**Figure 1B**). The aortic endothelium and smooth muscle from IRSA-affected dams also showed increased staining for TGF-β1 compared to controls (**Figure 1B**). In summary, aortic smooth muscle tissue from dams with *P. gingivalis* A7UF-induced IRSA showed an enhanced expression of TGF-β1 and endoglin that are implicated in vascular dysfunction (Ma et al., 2000; Tang et al., 2011; Tian et al., 2017).

**Figure 2:**
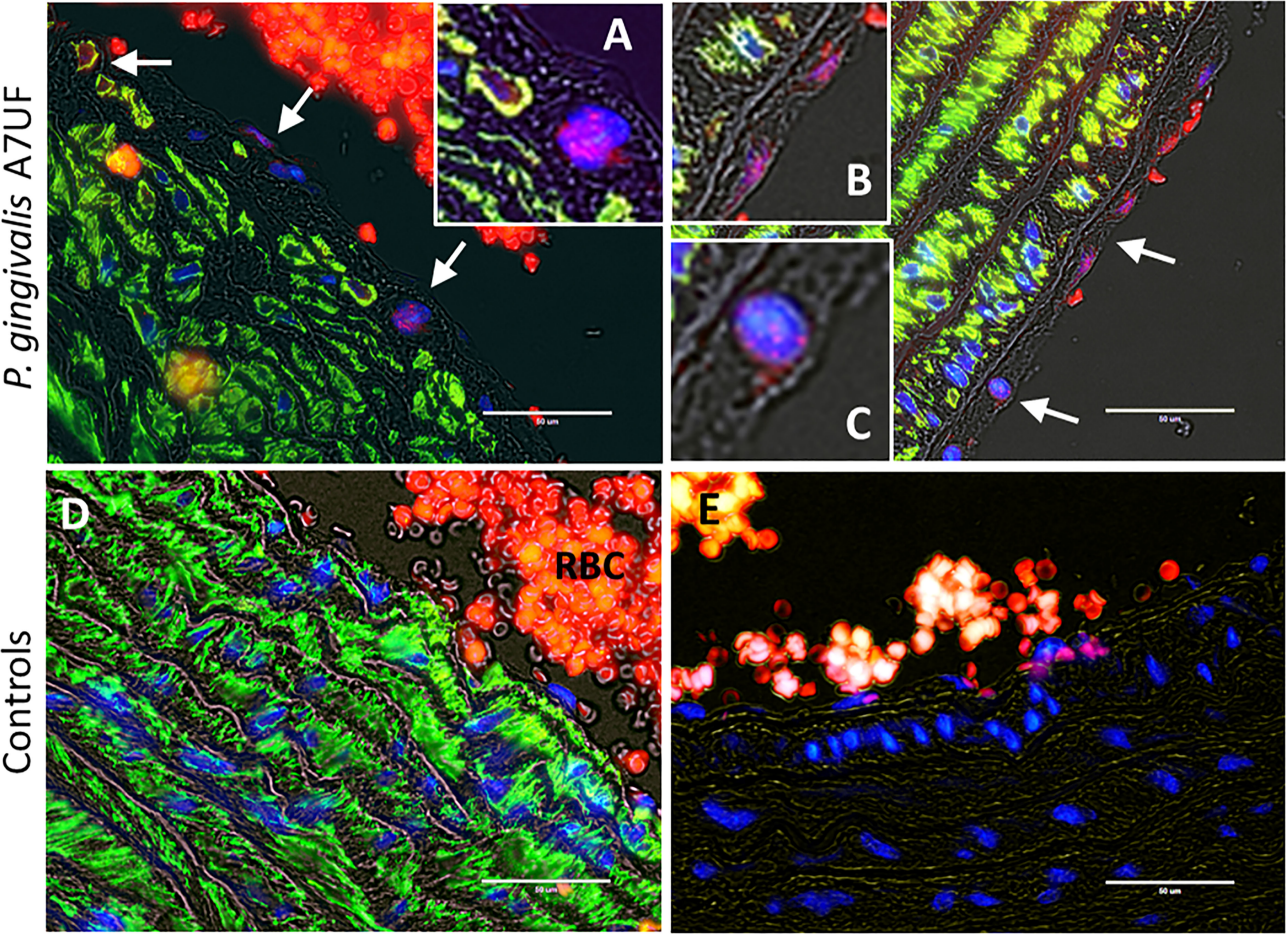
*In situ* detection of *P. gingivalis* in rat aortic specimens. White arrows indicate regions positive for *P. gingivalis* (red). VSMCs (green) were counterstained with an antibody to smooth muscle actin. Nuclei were stained with DAPI. **(A-C)** are magnified insets of regions positive for *P. gingivalis*. **(D)** Sham-inoculated control RBCs = red blood cells. **(E)** Isotype control. Transillumination (gray) was used to delineate tissue architecture. Scale bar = 50 µm.

Since our IRSA model depends on a chronic infection with *P. gingivalis* A7UF, rat aortic arch and valve specimens from the same animals examined in **Figure 1** were also examined for colonization by *P. gingivalis* (**Figure 2**). *P. gingivalis* protein was detected by immunofluorescent histology. To identify AoSMCs, tissues were counterstained with an antibody to smooth muscle actin (ACTA). *P. gingivalis* A7UF was identified in aortic valve sections from three of six infected dams. *P. gingivalis* protein was detected in AoSMCs adjacent to the intima (**Figure 2A**), and the aggregates of *P. gingivalis* were found most often in the aortic intima in association with endothelium and ACTA-negative cells (**Figures 2A-C**). *P. gingivalis* A7UF was not detected in any aortic specimens from sham-inoculated controls (**Figure 2D**).

### *P. gingivalis* A7UF and W83 induce different *in vitro* responses in rat aortic smooth muscle cells

**Figure 3:**
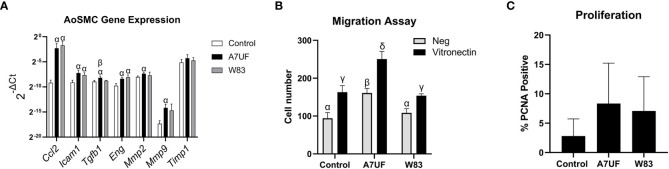
Impact of the *in vitro* infection of AoSMCs with *P. gingivalis*. **(A)** Aortic smooth muscle (AoSMC) gene expression following infection with A7UF or W83. Values represent the mean ± SD (n = 5) from two independent experiments. Bars labeled with α indicate values are significantly different from sham control (P < 0.05). The A7UF bar labeled with β indicates that this value is also significantly different from W83 (P < 0.05). **(B)** AoSMC migration assessed by the Transwell assay. Values represent the mean ± SD of (n = 4) from two independent experiments. α, β, γ, and δ indicate that the value is significantly different from all other groups (P < 0.05). **(C)** AoSMC proliferation assessed by proportion of PCNA-positive nuclei. Values represent the mean ± SD of (n = 6).

We next determined whether *in vitro* infection with IRSA-inducing strain A7UF and/or nonIRSA-inducing strain W83 recapitulates VSMC dysfunction observed with IRSA *in vivo*. AoSMCs were infected *in vitro* with A7UF or W83 and assessed for phenotype switching (**Figure 3**). Infection with A7UF induced an increased expression of *Ccl2*, *Icam1*, *Tgfb1*, *Eng*, and matrix metalloproteinase (*Mmp*) 2 and 9 with no change in the expression of the tissue inhibitor of metalloproteinase (*Timp*) (**Figure 3A**). In contrast, infection with W83 only increased the AoSMC expression of *Ccl2*, *Icam1*, and *Eng* (**Figure 3A**). When comparing gene expression profiles between A7UF and W83, only *Tgfb1* expression was significantly increased in the A7UF group (**Figure 2A**). Infection with A7UF also enhanced AoSMC migration before and after the addition of the agonist vitronectin (**Figure 3B**). Infection with W83 had no significant effect on AoSMC migration compared to control cells (**Figure 2B**). The infection of AoSMCs with either A7UF or W83 had no significant effect on AoSMC proliferation (**Figure 3C**). In summary, A7UF induced phenotypic changes in AoSMCs that can impact vascular remodeling (i.e., increased migration and expression of *Tgfb1*, *Mmp2*, and *Mmp9*), which were not observed in W83-infected cells.

### *P. gingivalis* A7UF and W83 bind differently to rat aortic proteins

**Figure 4:**
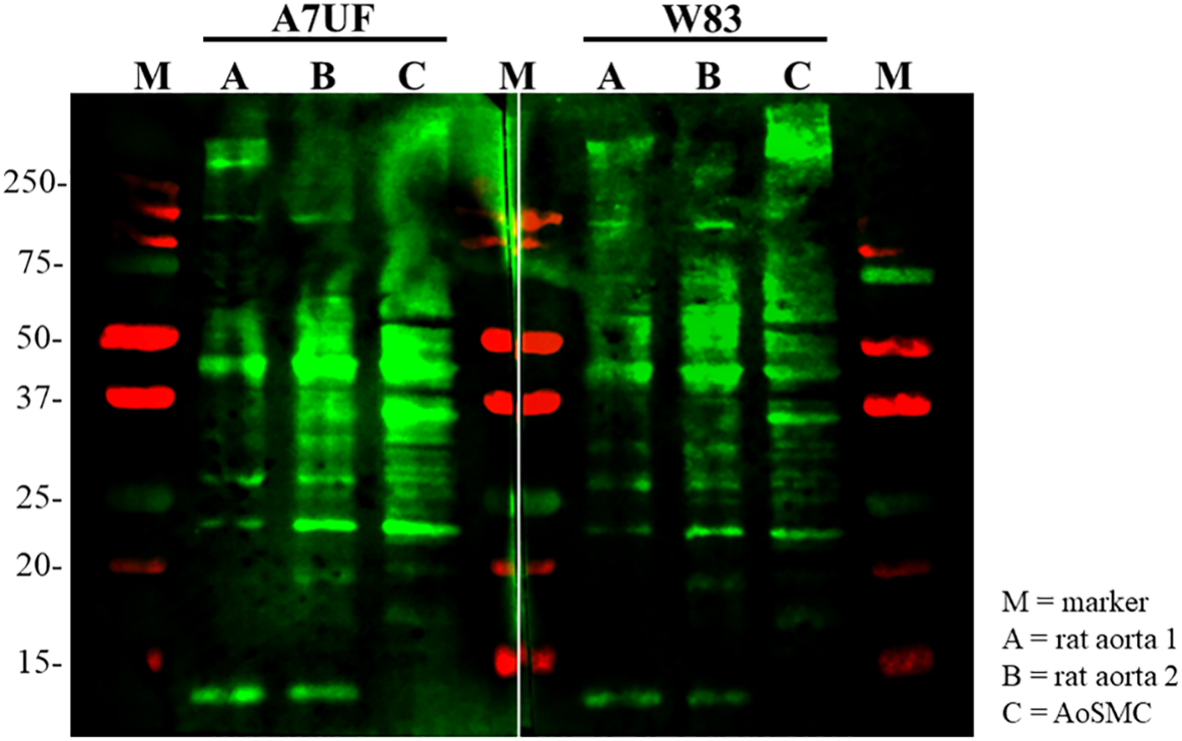
Far-Western blot showing the differential binding pattern of *P. gingivalis* A7UF and W83 to rat aortic tissue and AoSMCs. Far-western image is representative of two independent experiments. Numbers indicate marker reference sizes in kDa.

After establishing that A7UF and W83 have different effects on AoSMC phenotype switching *in vitro*, we next determined if proteins from *P. gingivalis* A7UF and W83 bound differently to rat aortic tissue and AoSMCs by far-Western blot. Both A7UF and W83 proteins bound to rat aortic proteins with some similarity. However, more A7UF proteins bound to rat proteins in the 20–40 kDa size than W83. In contrast, more W83 proteins bound to rat proteins in the 50–250 kDa size than A7UF (**Figure 4**).

### *P. gingivalis*/aortic smooth muscle cell protein interactions by pull-down assay

Since our objective was to identify bacterial proteins that are most likely to affect cell signaling events in VSMCs (i.e., membrane receptors), we used AoSMC membrane proteins as the bait in our pull-down assays. However, prey protein extracts included all soluble bacterial compartments (cytosol and membrane associated). LC/MS/MS identified 490 proteins in the A7UF eluate; of these, 232 proteins were also found in the W83 eluate (**Supplement File 2**). Based on their database annotation and cross-referenced against experimentally supported reports, these captured prey proteins localize to the cytosol, inner membrane, periplasmic space, outer membrane, and outer surface or are secreted. Since our interest was in bacterial proteins that would directly interact with the host cell during natural infections, the presumptive cytosolic proteins identified in this assay were excluded from further consideration in our initial screening.

Spectral counting was used to quantify and compare each protein in each sample. To reduce the risk of considering artifactual interactions, we set our threshold level for the screening and identification of strain-specific non-cytosolic proteins captured in the pull-down assay eluates to a minimum of 10 or more spectral counts. Based on these criteria, we identified eight membrane-associated or secreted proteins in the A7UF-only eluate (**Table 1**). None of the proteins present in the W83 eluate met our criteria (i.e., 10 or more spectral counts). Only two proteins were identified as unique to the W83 eluate, annotated as uncharacterized proteins (PG1507 and PG0987); however, their spectral counts were 4 and 6, respectively.

**Table 1:** Proteins only detected in the A7UF-eluted fraction with 10 or more spectral counts.

ID^A^	Location in *P. gingivalis* cell (predicted or known function)	Counts^B^
PG0724	Dipeptidyl-peptidase 5 (periplasmic enzyme, amino acid metabolism)	41
PG0809	Sov—T9SS outer membrane surface (bacterial motility and gingipain secretion)	26
PG0026	PorU—T9SS outer membrane surface (sortase)^C^	15
PG0534	PorF T9SS outer membrane	15
PG2216	Uncharacterized outer membrane protein (T9SS cargo; putative carbohydrate hydrolysis)	10
PG1430	TPR domain membrane protein (protein complex assembly)	10
PG2102	TapA—immunoreactive 61 kDa antigen, aka. PG91 (T9SS cargo) ^C^	10
PG2155	Membrane lipoprotein (putative metalloprotease/endopeptidase)	10

**A)** ID = W83 gene locus or gene name.

**B)** Spectral counts detected in IRSA-inducing A7UF.

**C)** CTD c-terminal sorting domain/por_secre_tail identified in the UniProt database.

**Figure 5:**
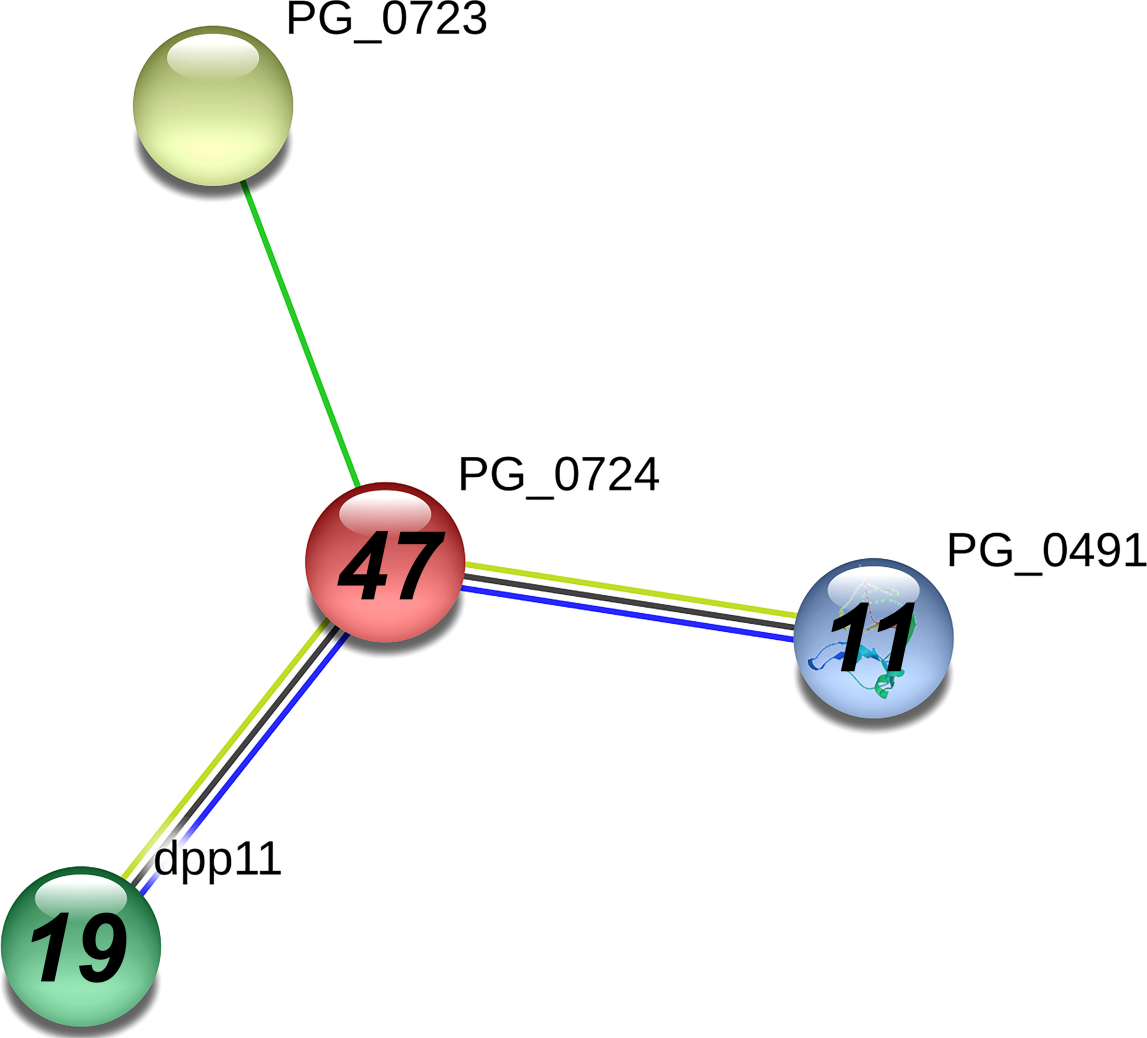
PG_0724 interaction network using STRING database.org. Network nodes represent all proteins produced by a single, protein-coding *P. gingivalis* W83 gene locus. Bold text in nodes indicates the spectral counts of the protein found in the A7UF eluate. Italicized numbers within nodes indicate the fold greater number of spectral counts in the A7UF eluate compared to W83. Nodes without numbers indicate proteins that were not identified in the pull-down eluates. Protein interactions include neighborhood (bright green), text-mining (pale green), coexpression (black), and gene co-occurrence (blue).

In **Table 1**, dipeptidyl-peptidase 5 (PG0724), a periplasmic enzyme involved in amino acid metabolism, had the highest spectral counts (41) in the A7UF eluate that were undetected in the W83 eluate. The STRING analysis of PG0724 set at high confidence (≥0.700) showed an association of this protein with two other dipeptidyl-peptidases, dpp11(PG1283) and dpp7 (PG0491), both of which were detected in higher concentrations in the A7UF eluate (**Figure 5**). An uncharacterized protein, PG0723, was not detected in the pull-down eluates.

**Figure 6:**
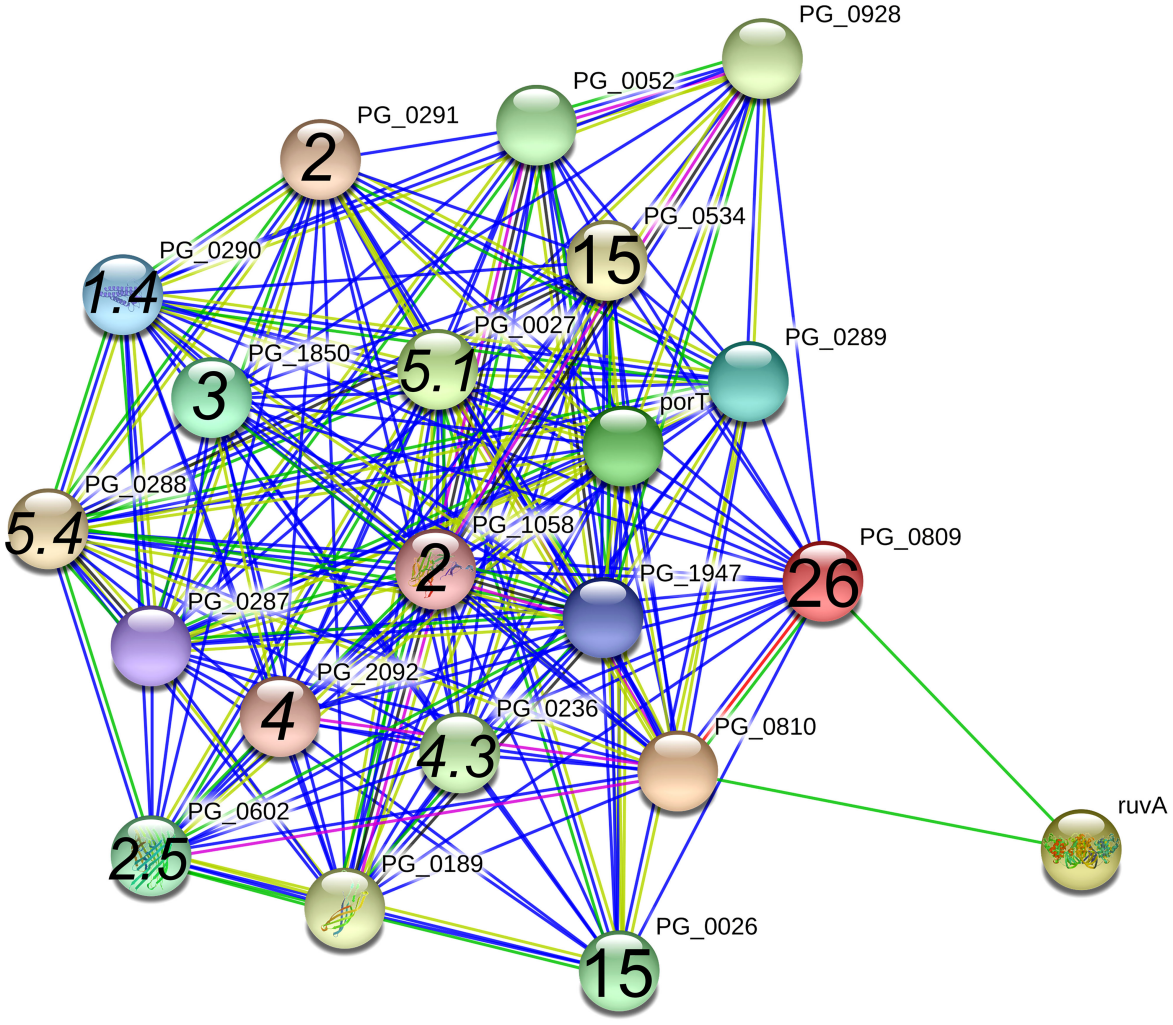
PG_0809 interaction network using STRING database.org. Network nodes represent all proteins produced by a single, protein-coding *P. gingivalis* W83 gene locus. Bold text in nodes indicate the spectral counts of the protein found in the A7UF eluate. Italicized numbers within nodes indicate the fold-greater number of spectral counts in the A7UF eluate compared to W83. Nodes without numbers indicate proteins that were not identified in the pull-down eluates. Protein interactions include experimentally determined (pink), gene fusions (red), neighborhood interactions (bright green), text-mining (pale green), and gene co-occurrence (blue).

PG0809, also known as Sov, which is a component of the type 9 secretion system (T9SS), had the next highest number of spectral counts in the A7UF eluate. The STRING protein interaction analysis of PG0809, which was set at high confidence (≥0.700), listed several proteins that are the components of the T9SS, its cargo, or others (**Figure 6**) (Heath et al., 2016; Lasica et al., 2017). Of these, PG0026, and PG0534 are listed in **Table 1**. The other known T9SS component proteins identified by STRING (**Figure 6**) and detected in the pull-down assay included PG1058 (A7UF eluate alone) and others that were in higher concentration in the A7UF eluate including PG0027, PG0236, PG0288, PG0290, PG0291, PG0602, PG1850, PG2092 and PG2172 (**Table 2**). Proteins identified by STRING (**Figure 6**) but not detected in the pull-down eluates included PorT (T9SS outer membrane component); PG0052 (PorY, T9SS sensor histidine kinase); PG0189 (PorG, T9SS outer membrane component), PG0287 (PorP, T9SS outer membrane component), PG0289 (PorL, T9SS inner membrane component); PG0810 (uncharacterized protein); PG0928 (PorX, T9SS response regulator); PG1947 (PorW, T9SS periplasmic component); and ruvA (Holliday junction ATP-dependent DNA helicase). PG1786 is another T9SS component (Heath et al., 2016) that was not detected by STRING analysis but was present in the pull-down eluates (**Tables 2**, **S1**).

**Table 2:** T9SS components detected in the A7UF-only eluate or both.

ID^A^	Protein description	Counts^B,C^
PG0027	PorV (LptO)—outer membrane	5.1^C^
PG0236	Right-handed β-helix region/putative carbohydrate hydrolysis domain (pectin_lyase_fold/virulence domain) periplasmic protein; hypothetical T9SS component^E^	4.3^C^
PG0288	PorK—periplasmic lipoprotein	5.4^C^
PG0290	PorM—periplasmic lipoprotein (T9SS complex motor)	1.4^C^
PG0291	PorN—periplasmic (interacts with PorP, PorK, PorL, and PorM)	2^B^
PG0602	PorQ—outer membrane	2.5^C^
PG1058	PorE—periplasmic lipoprotein (T9SS complex assembly); immunoreactive 75 kDa protein (aka. PG4)	2^B^
PG1604	PorZ—outer surface; immunoreactive 84 kDa antigen (aka PG93)^D^	1.8^C^
PG1786	DUF3078 outer membrane protein; hypothetical T9SS component^E^	2^C^
PG1850	DUF4835 periplasmic protein; hypothetical T9SS component^E^	3^B^
PG2092	Plug; DUF5103 periplasmic protein; hypothetical T9SS component^E^	4^C^
PG2172	PorA; putative T9SS component and outer membrane relay for the PorXY-SigP signaling system^E^	2.2^C^

**A)** ID = W83 gene locus or gene name.

**B)** Spectral counts detected in IRSA-inducing A7UF.

**C)** Ratio of spectral counts A7UF/W83.

**D)** CTD c-terminal sorting domain/por_secre_tail identified in the UniProt database.

**E)** Reported in Heath et al. (2016).

Other *P. gingivalis* proteins that were only detected in the A7UF eluate (**Table 1**) included two T9SS substrate uncharacterized outer membrane proteins: PG2216 and PG2102 (known as an immunoreactive 61 kDa antigen) (Veith et al., 2014). The other proteins listed in **Table 1** were PG1430, an uncharacterized membrane protein that contains a TPR domain putatively involved in protein complex assembly, and PG2155, an uncharacterized membrane protein that contains a metalloprotease domain. The STRING interaction maps for these proteins can be found in the **Supplement File 1**.

We next screened proteins present in both A7UF and W83 eluates. Because our interest was in membrane proteins that were enriched in the A7UF sample, we normalized spectral counts by dividing the A7UF counts by W83 and set the cut-off value as positive for enrichment at threefold or greater in the A7UF eluate. Based on this criteria, additional 16 T9SS components or cargo proteins were identified (**Table 3**). Known virulence factors, RgpB gingipains (4.2-fold greater in A7UF), HagE/RgpA gingipains (3.2-fold greater in A7UF), CPG70 zinc carboxypeptidase (3.2-fold greater in A7UF), and mfa5 minor fimbrial subunit (4-fold greater in A7UF), were included in this group. Other proteins that fit this criteria but were not T9SS substrates included HmuR, (PG1552, Ton-B dependent hemoglobin receptor, 6.4-fold greater in A7UF), PG2008 (Ton-B dependent receptor, 3.3-fold greater in A7UF), and PG1835 (surface lipoprotein, 3-fold greater in A7UF) (**Table S1**).

**Table 3:** T9SS cargo proteins detected in the A7UF-only eluate or both.

ID^A^	Protein description	Counts^B,C^
PG0026	PorU; surface C-terminal signal peptidase	15^B^
PG0182	Mfa5; minor fimbrial subunit/Von Willebrand type A domain^E^	4^C^
PG0183	Membrane lipoprotein^E^	7^C^
PG0232	Immunoreactive 92 kDa antigen (aka. PG21); putative CPG70 zinc carboxypeptidase outer membrane protein^DE^	3.2^C^
PG0350	Internalin related; LRR domain outer membrane protein^E^	2.6^C^
PG0410	Uncharacterized protein with a peptidase domain^D^	2.1^C^
PG0411	Hemaglutinnin—outer membrane protein^DE^	2.1^C^
PG0495	Uncharacterized protein^DE^	3.6^C^
PG0506	RgpB; arginine-specific gingipains—outer membrane protein^DE^	4.2^C^
PG0553	Putative lysine-specific endopeptidase outer membrane protein^DE^	3.3^C^
PG0611	Periplasmic lipoprotein^E^	3.4^C^
PG0614	Uncharacterized protein^E^	11^C^
PG0616	HBP35 thioredoxin—outer membrane protein^DE^	2.3^C^
PG0626	Uncharacteried protein—outer membrane protein^DE^	6^C^
PG0654	Uncharacteried protein—outer membrane protein^DE^	1.6^C^
PG1030	Uncharacterized periplasmic protein^DE^	2.4^C^
PG1035	Uncharacterized membrane protein; CHU_C secretion signal domain	3^B^
PG1374	Immunoreactive 47 kDa antigen (aka. PG97); LRR domain—outer membrane protein^DE^	2.9^c^
PG1424	Peptidylarginine deiminase (PPAD)—outer membrane protein^DE^	2.2^C^
PG1604	PorZ; Immunoreactive 84 kDa antigen (aka. PG93) T9SS component^DE^	1.8^C^
PG1795	Uncharacterized outer membrane protein^DE^	8^C^
PG1798	Immunoreactive 46 kDa antigen (aka. PG99)^DE^	4.2^C^
PG1837	HagA; hemagglutinin A—outer membrane protein^E^	2.9^C^
PG2024	HagE/RgpA (nemagglutinin E; arginine-specific gingipain)	3.2^C^
PG2102	TapA; immunoreactive 61 kDa antigen (aka PG91)—outer membrane protein^DE^	10^B^
PG2172	PorA; putative outer membrane relay for the PorXY-SigP signaling system (FimH-like mannose binding domain, SWIM-type domain)^DE^	2.2^C^
PG2216	Uncharacterized outer membrane protein with a putative T9SS targeting domain (DUF6383) and a putative carbohydrate hydrolysis domain (pectin_lyase_fold/virulence domain)^E^	10^C^

A) ID = W83 gene locus or gene name.

B) Spectral counts detected in IRSA-inducing A7UF.

C) Ratio of spectral countsA7UF/W83 rounded to the next whole number.

D) CTD c-terminal sorting domain/por_secre_tail identified in uniprot database.

E) Predicted CTD c-terminal sorting domain (Veith et al., 2013) and/or identified as substrates of T9SS (Glew et al., 2012; Heath et al., 2016).

### *P. gingivalis* A7UF and W83 proteome profiles

To determine if results of the pull-down assay were caused by differences in the A7UF and W83 proteome, we profiled the proteins present in the protein extracts that were used for the pull-down assay (**Supplement File 2**, **Table S2**). Overall, most proteins detected in the pull-down assay were present in both A7UF and W83 proteomes. We first screened the A7UF and W83 proteomes for the proteins that were only detected in the A7UF pull-down eluate (**Table 1**). The spectral counts from PG0724 (Dpp5), PG0809 (T9SS component Sov), PG0026 (T9SS component PorU), PG2216 (T9SS cargo), and PG1430 (TPR domain protein) were enriched in the W83 proteome compared to A7UF (**Supplement File 2**, **Table S3**); these proteins had the highest spectral counts in the A7UF pull-down eluate (**Table 1**). In contrast, PG0534 (PorF) and PG2155 were more abundant in the A7UF proteome than W83 (7.7 spectral counts vs. 2.9). Thus, despite these proteins not being captured and detected in the W83 pull-down assay, Sov, PorU, and PorF were present in the W83 protein lysate that was used as bait.

We then screened the proteome profiles of A7UF and W83 for all T9SS components (**Supplement File 2**, **Table S3**). PG0182 (mfa5) was not detected in either the A7UF or W83 proteome. In total, 16 of the T9SS cargo proteins detected in the pull-down assay were enriched in the W83 proteome. These were PG0232, PG0410, PG0411, PG0506, PG0611, PG0626, PG0654, PG1030, PG1035, PG1424, PG1604, PG1798, PG1837, PG2172, and PG2216. The T9SS cargo proteins that were enriched in the A7UF proteome included PG0183, PG0350, PG0495, PG0553, PG0614, PG0616, PG1374, PG1795, PG2003, and PG2102. PorX (PG0928), which is the cytoplasmic response regulator of T9SS expression detected in both proteomes, was not detected in the pull-down eluates. However, PorA (PG2172), the putative outer membrane relay protein of the PorXY-SigP signaling pathway (Yukitake et al., 2020, Gorasia et al., 2022) was detected in the pull-down eluate. PorX was enriched in the A7UF proteome, but PorA was enriched in the W83 proteome. Neither the inner membrane sensor kinase PorY (PG0052) nor the cytoplasmic SigP (PG0162) sigma factor components of the PorXY-SigP regulatory system for T9SS expression were detected in either the proteome or eluate.

## Discussion

Our objective was to discover *P. gingivalis* virulence factors operating at the bacterial/host interface that could be responsible for VSMC dysfunction. To increase the likelihood that we identified VSMC/*P. gingivalis* protein interactions specific to IRSA, nonIRSA-inducing W83 was used as a VSMC phenotype negative control. Because of the technical challenges that prevent isolating purified VSMCs from the placental bed, it was necessary to determine if rat AoSMCs could serve as a surrogate for spiral arterial smooth muscle. We first established that dams with *P. gingivalis* A7UF-induced IRSA also show AoSMC changes along with the *P. gingivalis* colonization of the tissue. Moreover, the *in vitro* infection of AoSMCs with IRSA-inducing strain A7UF showed similar changes in AoSMC responses to infection that were distinct from infection by non-IRSA-inducing strain W83. After validating that our approach was sound, the pull-down assay detected various membrane-associated proteins unique to or enriched in the IRSA-inducing A7UF fraction.

The most prominent and biologically compelling group of proteins that were enriched in the A7UF pull-down eluate are the components of the T9SS and its cargo. It has already been established that the T9SS plays an essential role in the processing and export of major virulence factors including gingipains and peptidylarginine deiminase (Glew et al., 2012; Sato et al., 2013) where they can interact with the host cell during bacterial attachment or through outer membrane vesicles (Veith et al., 2014). Among the 33 T9SS substate proteins (cargo) reported by Health et al. (Heath et al., 2016), 25 of them were detected as interacting with AoSMCs and were enriched for in the IRSA-inducing strain A7UF relative to W83, supporting the hypothesis that they play a leading role in host–pathogen interactions. Given that other bacterial secretion systems such as T3SS, T4SS, and T6SS transport bacterial proteins across cell membranes (Green and Mecsas, 2016), the high affinity of T9SS components for host cells implies that the *P. gingivalis* T9SS may serve a similar function during *in vivo* infection.

A further support of the biological relevance of our data was the identification of known immunoreactive proteins. Among the enriched T9SS components and substrates identified, six (PG0232, PG1058, PG1374, PG1604, PG1798, and PG2102) have been previously reported as immunoreactive *in vivo* and confirmed using a murine subcutaneous abscess model (Ross et al., 2009). The additional reportedly immunoreactive proteins that interacted with AoSMCs in our pull-down assays include four outer membrane proteins (**Table S1**): PG0668 (putative TonB-dependent ligand-gated receptor), PG2054 (Porin F-like periplasmic lipoprotein), PG2167 (TPR domain PgmA paralog that complexes with PG2168), and PG2173 (Omp28 lipoprotein) (Veith et al., 2021). The only T9SS substrate previously reported as immunoreactive that was not detected in either the proteome or the pull-down assay was PG2100 (TapC), an outer membrane protein of unknown function found in W83 but not *P. gingivalis* reference strain 33277.

Our study does not establish causality between the T9SS/AoSMC interaction and VSMC dysfunction. However, RgpB gingipains, which is a T9SS cargo protein that was enriched in the A7UF eluate, affects several host cell pathways that regulate VSMC plasticity such as the focal adhesion pathway (involved in cell migration), NOD-like receptor signaling pathway, MAPK signaling pathway modulates the downstream effectors of TGF-β signaling), and TGF-beta signaling pathway (Zhang et al., 2016). The Mfa5 minor fimbrial subunit that was also enriched in the A7UF eluate may also affect VSMCs since it is proposed to act as a ligand for host cell receptors whereby *P. gingivalis* could subvert host cell signaling (Hasegawa and Nagano, 2021).

The potential impact of the other proteins that were only detected in the A7UF pull-down eluate (**Table 1**) deserve mention. Very little is known about the potential virulence of *P. gingivalis* dipeptidyl-peptidase 4. Dipeptidyl-peptidase plays a role in nutrient acquisition and, in that sense, may facilitate bacterial survival and long-term colonization within the host (Nemoto and Ohara Nemoto, 2021).

Our method of using *P. gingivalis* whole cell lysates as prey in our pull-down assay resulted in numerous AoSMC/*P. gingivalis* interactions that seem biologically irrelevant during active colonization. Such examples include bacterial proteins that are normally confined to the cytosol and inner membrane that were present in both A7UF and W83 eluates. Our pull-down assay also captured periplasmic proteins that were not expected to interact with AoSMCs based on the same reasoning. However, in the case of periplasmic T9SS components such as PorK, PorN, and PorM, we wondered if their presence in the A7UF eluate was due to their binding to T9SS-interacting partners rather than a direct interaction with AoSMCs.

Comparing the proteome profiles of the “prey” protein extracts to the pull-down eluates provided additional insights. First, it clarified that negative results in the W83 pull-down eluate was not due to the lack of protein expression in W83. On the contrary, several proteins that were unique to or enriched in the A7UF pull-down were in higher abundance in the W83 prey protein preparation (proteome). Therefore, other factors such as post-translational modifications of *P. gingivalis* proteins that could affect the stability of the bacterial protein to the host protein bond need to be considered. This phenomenon is particularly relevant to the T9SS cargo proteins including PorU that undergoes extensive glycosylation after the c-terminus is removed by the T9SS (Shoji et al., 2011; Gorasia et al., 2015). Protein glycosylation affects protein folding, targeted transport, cellular localization, and protein activity (Lin et al., 2020). Alternatively, there may be stability issues regarding the anchoring of the T9SS outer membrane complex to *P. gingivalis* LPS in W83. For example, the W83 clone that we used in our study is K-antigen capsule negative (Kim et al., 2021). This may explain why components that form the attachment complex, PorU/PorV/PorQ/PorZ (Gorasia et al.), were missing or decreased in the W83 pull-down eluate despite PorU, PorV, and PorZ being in greater abundance in the W83 prey preparation. It is unlikely that the results of the pull-down assay were caused by variability in our methodology since both *P. gingivalis* strains were cultured at the same time, in the same medium, and processed the same way. Further, the same concentration of W83 and A7UF protein lysates were loaded onto the affinity columns that contained the same AoSMC bait preparations within the same pull-down experiment. Finally, to avoid false-positive results, we optimized our assay to detect the strongest, most stable host/bacterial protein interactions.

It is not unusual that we observed a difference in the relative abundance of T9SS components in the A7UF and W83 protein extracts. Using proteomics, others have reported that the stoichiometric profiles of T9SS components differ between wildtype *P. gingivalis* W50 and 33277 strains (Gorasia et al., 2020). However, unlike Gorasia et al. (2020), who detected all the components of the T9SS in their preparations, we did not. Compared to conventional LC-MS/MS coupled to the UHPLC of the in-gel digests of whole cells as used by Gorasia et al. (2020), our proteome profiles were generated using NanoLC-MS/MS coupled to a NanoHPLC of liquid protein digests at high-resolution gradient length, which typically results in low-abundance peptides dropping below the limit of detection. Thus, the lack of detection of certain proteins in the proteome assay (e.g., those captured in the pull-down assay for both W83 and A7UF indicating that they were present in the protein preparations) was a result of the relatively low abundance of identifiable peptides. Further, our methodology required physiologic buffer conditions for optimal protein–protein interactions, which would result in the inevitable loss of insoluble *P. gingivalis* proteins in our preparation. This is a limitation of this approach, and, as such, there is a risk that we have not captured all *P. gingivalis* proteins capable of binding to AoSMCs. Another limitation of our study is that we used one set of bacterial culture conditions that would not reflect the full repertoire of *P. gingivalis* proteomes that change with the composition of the growth medium (Veith et al., 2018). However, an important caveat of our approach was that it was designed to mimic conditions that we have observed in our *in vivo* model of infection (Phillips et al., 2018; Tavarna et al., 2020; Tavarna et al., 2022). Despite the limitations of our proteome study, we identified several *P. gingivalis* proteins in IRSA-inducing A7UF that had strong, stable interactions with AoSMC proteins that did not occur with non-IRSA-inducing W83. In addition, there is circumstantial evidence that proteins enriched in the A7UF pull-down eluate, such as T9SS cargo proteins RgpB and Mfa5 that affect host cell functions may affect vascular dynamics (Zhang et al., 2016; Hasegawa and Nagano, 2021), merit further study.

During the past decade, numerous studies have identified the components of the T9SS as well as its contribution to virulence in the form of processing and regulating the secretion of putative virulence factors (Sato et al., 2005; Sato et al., 2010; Shoji et al., 2011; Sato et al., 2013; Gorasia et al., 2015; Heath et al., 2016; Lasica et al., 2016; Taguchi et al., 2016; Glew et al., 2017; Madej et al., 2021). It was recently demonstrated that the T9SS modulates cytokine responses in macrophages and gingival fibroblasts (Braun et al., 2022). This study shows an association between the binding of the outer components of the *P. gingivalis* T9SS to VSMCs and their perturbed plasticity. The possibility that the T9SS is involved in the microbial manipulation of host cell signaling events important for cell differentiation and tissue remodeling would constitute a new virulence function for this system.

## Data availability statement

The datasets presented in this study can be found in online repositories. The names of the repository/repositories and accession number(s) can be found in the article/**Supplementary Material**.

## Ethics statement

The animal study was reviewed and approved by the University of Wisconsin Institutional Animal Care and Use Committee (protocol #V005576).

## Author contributions

PLP, X-JW and LR conceived the project. X-JW assisted with tissue collection, cell isolation, and proteome studies. PLP performed the bioinformatics analysis of identified proteins. LR and PLP wrote the manuscript. All authors contributed to the article and approved the submitted version.

## Funding

Research reported in this publication was supported by the Eunice Kennedy Shriver National Institute of Child Health and Human Development of the National Institutes of Health under Award NICHD-R03HD087633-01A1 and University of Wisconsin Fall Competition award. The content is solely the responsibility of the authors and does not necessarily represent the official views of the National Institutes of Health or The University of Wisconsin-Madison.

## Acknowledgments

We would like to thank Dr. Ann Progulske-Fox for the gift of *P. gingivalis* strain A7UF, Hong-Di Liu for his technical assistance with far-Western blotting and affinity chromatography, Sarah Malina and Duylinh Nguyen for their technical assistance with the *in vitro* characterization of infected AoSMC, and Gregorz Sabat and the Mass Spectrometry Core Facility, Biotechnology Center, University of Wisconsin-Madison for their services.

## Conflict of interest

The authors declare that the research was conducted in the absence of any commercial or financial relationships that could be construed as a potential conflict of interest.

## Publisher’s note

All claims expressed in this article are solely those of the authors and do not necessarily represent those of their affiliated organizations, or those of the publisher, the editors and the reviewers. Any product that may be evaluated in this article, or claim that may be made by its manufacturer, is not guaranteed or endorsed by the publisher.

## References

[B1] Barak S. Oettinger-Barak O. Machtei E. E. Sprecher H. Ohel G. (2007). Evidence of periopathogenic microorganisms in placentas of women with preeclampsia. J. Periodontol. 78, 670–676. doi: 10.1902/jop.2007.060362 17397314

[B2] Benagiano M. Mancuso S. Brosens J. J. Benagiano G. (2021). Long-term consequences of placental vascular pathology on the maternal and offspring cardiovascular systems. Biomolecules 11, 1–23. doi: 10.3390/biom11111625 PMC8615676 34827623

[B3] Braun M. L. Tomek M. B. Grünwald-Gruber C. Nguyen P. Q. Bloch S. Potempa J. S.. (2022). Shut-down of type IX protein secretion alters the host immune response to *Tannerella forsythia* and *Porphyromonas gingivalis* . Front. Cell Infect. Microbiol. 12, 835509. doi: 10.3389/fcimb.2022.835509 35223555 PMC8869499

[B4] Brosens I. Benagiano M. Puttemans P. D'elios M. M. Benagiano G. (2017). The placental bed vascular pathology revisited: a risk indicator for cardiovascular disease. J. Matern. Fetal. Neonatal. Med. 32, 1–9. doi: 10.1080/14767058.2017.1409718 29172831

[B5] Brosens I. Pijnenborg R. Vercruysse L. Romero R. (2011). The "Great obstetrical syndromes" are associated with disorders of deep placentation. Am. J. Obstet. Gynecol. 204, 193–201. doi: 10.1016/j.ajog.2010.08.009 21094932 PMC3369813

[B6] Brown S. L. Lundgren C. H. Nordt T. Fujii S. (1994). Stimulation of migration of human aortic smooth muscle cells by vitronectin: implications for atherosclerosis. Cardiovasc. Res. 28, 1815–1820. doi: 10.1093/cvr/28.12.1815 7532547

[B7] Cao C. Ji X. Luo X. Zhong L. (2015). Gingipains from *Porphyromonas gingivalis* promote the transformation and proliferation of vascular smooth muscle cell phenotypes. Int. J. Clin. Exp. Med. 8, 18327–18334. 26770435 PMC4694335

[B8] Chaparro A. Blanlot C. Ramírez V. Sanz A. Quintero A. Inostroza C.. (2013). *Porphyromonas gingivalis*, *Treponema denticola* and toll-like receptor 2 are associated with hypertensive disorders in placental tissue: a case–control study. J. Periodontal Res. 48, 802–809. doi: 10.1111/jre.12074 23711357

[B9] Glew M. D. Veith P. D. Chen D. Gorasia D. G. Peng B. Reynolds E. C. (2017). PorV is an outer membrane shuttle protein for the type IX secretion system. Sci. Rep. 7, 8790. doi: 10.1038/s41598-017-09412-w 28821836 PMC5562754

[B10] Glew M. D. Veith P. D. Peng B. Chen Y. Y. Gorasia D. G. Yang Q.. (2012). PG0026 is the c-terminal signal peptidase of a novel secretion system of *Porphyromonas gingivalis* . J. Biol. Chem. 287, 24605–24617. doi: 10.1074/jbc.M112.369223 22593568 PMC3397888

[B11] Gorasia D. G. Glew M. D. Veith P. D. Reynolds E. C. (2020). Quantitative proteomic analysis of the type IX secretion system mutants in *Porphyromonas gingivalis* . Mol. Oral. Microbiol. 35, 78–84. doi: 10.1111/omi.12283 32040252

[B12] Gorasia D. G. Veith P. D. Chen D. Seers C. A. Mitchell H. A. Chen Y. Y.. (2015). *Porphyromonas gingivalis* type IX secretion substrates are cleaved and modified by a sortase-like mechanism. PloS Pathog. 11, e1005152. doi: 10.1371/journal.ppat.1005152 26340749 PMC4560394

[B13] Gorasia D. G. Veith P. D. Reynolds E. C. (2022). Protein interactome mapping of porphyromonas gingivalis provides insights into the formation of the PorQ-z complex of the type IX secretion system. Mol. Oral. Microbiol. 10, 1–7. doi: 10.1111/omi.12383 PMC10947112 35862235

[B14] Green E. R. Mecsas J. (2016). Bacterial secretion systems: An overview. Microbiol. Spectr. 4, 1. doi: 10.1128/microbiolspec.VMBF-0012-2015 PMC4804464 26999395

[B15] Harris L. K. Benagiano M. D’elios M. M. Brosens I. Benagiano G. (2019). Placental bed research: II. functional and immunological investigations of the placental bed. Am. J. Obstetrics Gynecol. 221, 457–469. doi: 10.1016/j.ajog.2019.07.010 31288009

[B16] Hasegawa Y. Nagano K. (2021). *Porphyromonas gingivalis* FimA and Mfa1 fimbriae: Current insights on localization, function, biogenesis, and genotype. Jpn Dent. Sci. Rev. 57, 190–200. doi: 10.1016/j.jdsr.2021.09.003 34691295 PMC8512630

[B17] Heath J. E. Seers C. A. Veith P. D. Butler C. A. Nor Muhammad N. A. Chen Y. Y.. (2016). PG1058 is a novel multidomain protein component of the bacterial type IX secretion system. PloS One 11, e0164313. doi: 10.1371/journal.pone.0164313 27711252 PMC5053529

[B18] Hermes W. Tamsma J. T. Grootendorst D. C. Franx A. van der Post J. Van Pampus M. G.. (2013). Cardiovascular risk estimation in women with a history of hypertensive pregnancy disorders at term: a longitudinal follow-up study. BMC Pregnancy Childbirth 13, 126. doi: 10.1186/1471-2393-13-126 23734952 PMC3680191

[B19] Hokamura K. Inaba H. Nakano K. Nomura R. Yoshioka H. Taniguchi K.. (2010). Molecular analysis of aortic intimal hyperplasia caused by *Porphyromonas gingivalis* infection in mice with endothelial damage. J. Periodontal Res. 45, 337–344. doi: 10.1111/j.1600-0765.2009.01242.x 19909399

[B20] Ibrahim M. I. Abdelhafeez M. A. Ellaithy M. I. Salama A. H. Amin A. S. Eldakrory H.. (2015). Can *Porphyromonas gingivalis* be a novel aetiology for recurrent miscarriage? Eur. J. Contracept Reprod. Health Care 20, 119–127. doi: 10.3109/13625187.2014.962651 25328050

[B21] Kim H. M. Ranjit D. K. Walker A. R. Getachew H. Progulske-Fox A. Davey M. E. (2021). A novel regulation of K-antigen capsule synthesis in *Porphyromonas gingivalis* is driven by the response regulator PG0720-directed antisense RNA. Front. Oral. Health 2. doi: 10.3389/froh.2021.701659 PMC8757827 35048039

[B22] Lasica A. M. Goulas T. Mizgalska D. Zhou X. De Diego I. Ksiazek M.. (2016). Structural and functional probing of PorZ, an essential bacterial surface component of the type-IX secretion system of human oral-microbiomic *Porphyromonas gingivalis* . Sci. Rep. 6, 37708. doi: 10.1038/srep37708 27883039 PMC5121618

[B23] Lasica A. M. Ksiazek M. Madej M. Potempa J. (2017). The type IX secretion system (T9SS): Highlights and recent insights into its structure and function. Front. Cell. Infection Microbiol. 7. doi: 10.3389/fcimb.2017.00215 PMC5445135 28603700

[B24] León R. Silva N. Ovalle A. Chaparro A. Ahumada A. Gajardo M.. (2007). Detection of *Porphyromonas gingivalis* in the amniotic fluid in pregnant women with a diagnosis of threatened premature labor. J. Periodontol. 78, 1249–1255. doi: 10.1902/jop.2007.060368 17608580

[B25] Lin B. Qing X. Liao J. Zhuo K. (2020). Role of protein glycosylation in host-pathogen interaction. Cells 9, 1–24. doi: 10.3390/cells9041022 PMC7226260 32326128

[B26] Liu W. Luo M. Zou L. Liu X. Wang R. Tao H.. (2019). uNK cell-derived TGF-β1 regulates the long noncoding RNA MEG3 to control vascular smooth muscle cell migration and apoptosis in spiral artery remodeling. J. Cell Biochem. 120, 15997–16007. doi: 10.1002/jcb.28878 31099432

[B27] Low E. L. Baker A. H. Bradshaw A. C. (2019). TGFβ, smooth muscle cells and coronary artery disease: a review. Cell. signalling 53, 90–101. doi: 10.1016/j.cellsig.2018.09.004 30227237 PMC6293316

[B28] Madej M. Nowakowska Z. Ksiazek M. Lasica A. M. Mizgalska D. Nowak M.. (2021). PorZ, an essential component of the type IX secretion system of *Porphyromonas gingivalis*, delivers anionic lipopolysaccharide to the PorU sortase for transpeptidase processing of T9SS cargo proteins. mBio 12, 1–14. doi: 10.1128/mBio.02262-20 PMC8545088 33622730

[B29] Ma X. Labinaz M. Goldstein J. Miller H. Keon W. J. Letarte M.. (2000). Endoglin is overexpressed after arterial injury and is required for transforming growth factor-beta-induced inhibition of smooth muscle cell migration. Arterioscler. Thromb. Vasc. Biol. 20, 2546–2552. doi: 10.1161/01.ATV.20.12.2546 11116051

[B30] Nankivell V. Primer K. Vidanapathirana A. Psaltis P. Bursill C. (2020). “Vascular biology of smooth muscle cells and restenosis,” in Mechanisms of vascular disease: A textbook for vascular specialists. Ed. Fitridge R. (Cham: Springer International Publishing).

[B31] Nemoto T. K. Ohara Nemoto Y. (2021). Dipeptidyl-peptidases: Key enzymes producing entry forms of extracellular proteins in asaccharolytic periodontopathic bacterium *Porphyromonas gingivalis* . Mol. Oral. Microbiol. 36, 145–156. doi: 10.1111/omi.12317 33006264 PMC8048996

[B32] Nesvizhskii A. I. Keller A. Kolker E. Aebersold R. (2003). A statistical model for identifying proteins by tandem mass spectrometry. Anal. Chem. 75, 4646–4658. doi: 10.1021/ac0341261 14632076

[B33] Park H. J. Kim Y. Kim M. K. Park H. R. Kim H. J. Bae S. K.. (2020). Infection of *Porphyromonas gingivalis* increases phosphate-induced calcification of vascular smooth muscle cells. Cells 9, 1–13. doi: 10.3390/cells9122694 PMC7765351 33334022

[B34] Phillips P. Brown M. B. Progulske-Fox A. Wu X. J. Reyes L. (2018). *Porphyromonas gingivalis* strain-dependent inhibition of uterine spiral artery remodeling in the pregnant rat. Biol. Reprod. 99, 1045–1056. doi: 10.1093/biolre/ioy119 29788108 PMC6297315

[B35] Ross B. C. Barr I. G. Patterson M. A. Agius C. T. Rothel L. J. Margetts M. B. (2009) Polypeptides and nucleotides. Available at: https://patents.justia.com/patent/8129500.

[B36] Sato K. Naito M. Yukitake H. Hirakawa H. Shoji M. Mcbride M. J.. (2010). A protein secretion system linked to bacteroidete gliding motility and pathogenesis. Proc. Natl. Acad. Sci. U.S.A. 107, 276–281. doi: 10.1073/pnas.0912010107 19966289 PMC2806738

[B37] Sato K. Sakai E. Veith P. D. Shoji M. Kikuchi Y. Yukitake H.. (2005). Identification of a new membrane-associated protein that influences transport/maturation of gingipains and adhesins of *Porphyromonas gingivalis* . J. Biol. Chem. 280, 8668–8677. doi: 10.1074/jbc.M413544200 15634642

[B38] Sato K. Yukitake H. Narita Y. Shoji M. Naito M. Nakayama K. (2013). Identification of *Porphyromonas gingivalis* proteins secreted by the por secretion system. FEMS Microbiol. Lett. 338, 68–76. doi: 10.1111/1574-6968.12028 23075153

[B39] Shoji M. Sato K. Yukitake H. Kondo Y. Narita Y. Kadowaki T.. (2011). Por secretion system-dependent secretion and glycosylation of *Porphyromonas gingivalis* hemin-binding protein 35. PloS One 6, e21372–e21372. doi: 10.1371/journal.pone.0021372 21731719 PMC3120885

[B40] Suwanabol P. A. Kent K. C. Liu B. (2011). TGF-beta and restenosis revisited: a smad link. J. Surg. Res. 167, 287–297. doi: 10.1016/j.jss.2010.12.020 21324395 PMC3077463

[B41] Swati P. Ambika Devi K. Thomas B. Vahab S. A. Kapaettu S. Kushtagi P. (2012). Simultaneous detection of periodontal pathogens in subgingival plaque and placenta of women with hypertension in pregnancy. Arch. Gynecol. Obstet. 285, 613–619. doi: 10.1007/s00404-011-2012-9 21830010

[B42] Taguchi Y. Sato K. Yukitake H. Inoue T. Nakayama M. Naito M.. (2016). Involvement of an skp-like protein, PGN_0300, in the type IX secretion system of *Porphyromonas gingivalis* . Infect. Immun. 84, 230–240. doi: 10.1128/IAI.01308-15 26502912 PMC4693996

[B43] Tang Y. Yang X. Friesel R. E. Vary C. P. H. Liaw L. (2011). Mechanisms of TGF-β-Induced differentiation in human vascular smooth muscle cells. J. Vasc. Res. 48, 485–494. doi: 10.1159/000327776 21832838 PMC3169366

[B44] Tavarna T. Phillips P. L. Wu X. J. Reyes L. (2020). Fetal growth restriction is a host specific response to infection with an impaired spiral artery remodeling-inducing strain of *Porphyromonas gingivalis* . Sci. Rep. 10, 14606. doi: 10.1038/s41598-020-71762-9 32884071 PMC7471333

[B45] Tavarna T. Wolfe B. Wu X. J. Reyes L. (2022). *Porphyromonas gingivalis*-mediated disruption in spiral artery remodeling is associated with altered uterine NK cell populations and dysregulated IL-18 and Htra1. Sci. Rep. 12, 14799. doi: 10.1038/s41598-022-19239-9 36042379 PMC9427787

[B46] Tian H. Ketova T. Hardy D. Xu X. Gao X. Zijlstra A.. (2017). Endoglin mediates vascular maturation by promoting vascular smooth muscle cell migration and spreading. Arteriosclerosis Thrombosis Vasc. Biol. 37, 1115–1126. doi: 10.1161/ATVBAHA.116.308859 PMC5444426 28450296

[B47] Vanterpool S. F. Been J. V. Houben M. L. Nikkels P. G. De Krijger R. R. Zimmermann L. J.. (2016). *Porphyromonas gingivalis* within placental villous mesenchyme and umbilical cord stroma is associated with adverse pregnancy outcome. PloS One 11, e0146157. doi: 10.1371/journal.pone.0146157 26731111 PMC4701427

[B48] Veerbeek J. H. Brouwers L. Koster M. P. Koenen S. V. Van Vliet E. O. Nikkels P. G.. (2016). Spiral artery remodeling and maternal cardiovascular risk: the spiral artery remodeling (SPAR) study. J. Hypertens. 34, 1570–1577. doi: 10.1097/HJH.0000000000000964 27219485

[B49] Veerbeek J. H. Hermes W. Breimer A. Y. Van Rijn B. B. Koenen S. V. Mol B. W.. (2015). Cardiovascular disease risk factors after early-onset preeclampsia, late-onset preeclampsia, and pregnancy-induced hypertension. Hypertension 65, 600–606. doi: 10.1161/HYPERTENSIONAHA.114.04850 25561694

[B50] Veith P. D. Chen Y. Y. Gorasia D. G. Chen D. Glew M. D. O'brien-Simpson N. M.. (2014). *Porphyromonas gingivalis* outer membrane vesicles exclusively contain outer membrane and periplasmic proteins and carry a cargo enriched with virulence factors. J. Proteome Res. 13, 2420–2432. doi: 10.1021/pr401227e 24620993

[B51] Veith P. D. Gorasia D. G. Reynolds E. C. (2021). Towards defining the outer membrane proteome of *Porphyromonas gingivalis* . Mol. Oral. Microbiol. 36, 25–36. doi: 10.1111/omi.12320 33124778

[B52] Veith P. D. Luong C. Tan K. H. Dashper S. G. Reynolds E. C. (2018). Outer membrane vesicle proteome of *Porphyromonas gingivalis* is differentially modulated relative to the outer membrane in response to heme availability. J. Proteome Res. 17, 2377–2389. doi: 10.1021/acs.jproteome.8b00153 29766714

[B53] Veith P. D. Nor Muhammad N. A. Dashper S. G. Likić V. A. Gorasia D. G. Chen D.. (2013). Protein substrates of a novel secretion system are numerous in the bacteroidetes phylum and have in common a cleavable c-terminal secretion signal, extensive post-translational modification, and cell-surface attachment. J. Proteome Res. 12, 4449–4461. doi: 10.1021/pr400487b 24007199

[B54] Villa-Bellosta R. Hamczyk M. R. (2015). Isolation and culture of aortic smooth muscle cells and *In vitro* calcification assay. Methods Mol. Biol. 1339, 119–129. doi: 10.1007/978-1-4939-2929-0_8 26445785

[B55] Whitley G. S. J. Cartwright J. E. (2010). Cellular and molecular regulation of spiral artery remodelling: Lessons from the cardiovascular field. Placenta 31, 465–474. doi: 10.1016/j.placenta.2010.03.002 20359743 PMC2882556

[B56] Yang W. W. Guo B. Jia W. Y. Jia Y. (2016). *Porphyromonas gingivalis*-derived outer membrane vesicles promote calcification of vascular smooth muscle cells through ERK1/2-RUNX2. FEBS Open Bio 6, 1310–1319. doi: 10.1002/2211-5463.12151 PMC5324769 28255538

[B57] Yukitake H. Shoji M. Sato K. Handa Y. Naito M. Imada K.. (2020). PorA, a conserved c-terminal domain-containing protein, impacts the PorXY-SigP signaling of the type IX secretion system. Sci. Rep. 10, 21109. doi: 10.1038/s41598-020-77987-y 33273542 PMC7712824

[B58] Zhang B. Elmabsout A. A. Khalaf H. Basic V. T. Jayaprakash K. Kruse R.. (2013). The periodontal pathogen *Porphyromonas gingivalis* changes the gene expression in vascular smooth muscle cells involving the TGFbeta/Notch signalling pathway and increased cell proliferation. BMC Genomics 14, 770. doi: 10.1186/1471-2164-14-770 24209892 PMC3827841

[B59] Zhang B. Sirsjo A. Khalaf H. Bengtsson T. (2016). Transcriptional profiling of human smooth muscle cells infected with gingipain and fimbriae mutants of *Porphyromonas gingivalis* . Sci. Rep. 6, 21911. doi: 10.1038/srep21911 26907358 PMC4764818

