# Supplementary material for: Differential affinity chromatography reveals a link between Porphyromonas gingivalis–induced changes in vascular smooth muscle cell differentiation and the type 9 secretion system
Source: Front Cell Infect Microbiol. 2022 Nov 22;12:983247. doi: 10.3389/fcimb.2022.983247 (PMC9722745; doi:10.3389/fcimb.2022.983247)
Supplement: Supplementary file 2 [file DataSheet_1.pdf]

Supplementary Figures

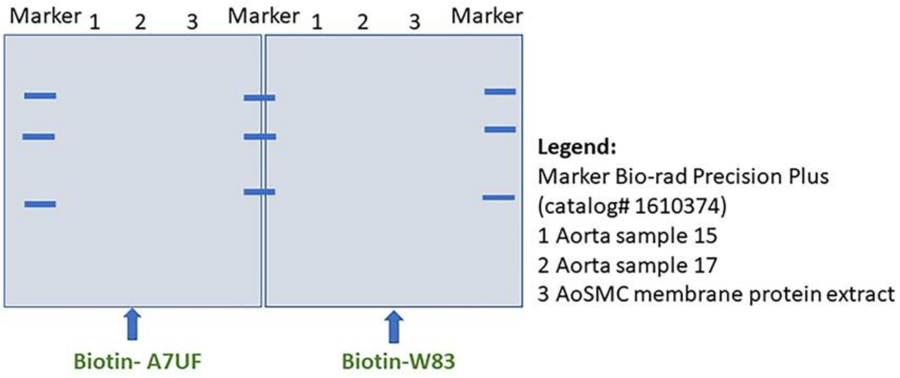

Figure. S1 Gel layout for the far-western blot. Equal amounts of each rat aorta whole protein extract (lanes 1 and 2) and membrane enriched AoSMC protein extract (lane 3) was loaded onto the same gel in duplicate. After protein transfer, the gel was cut in half before incubation biotin labeled A7UF or W83. The central marker lane was used to align both membranes for imaging with an Azure 600 Imaging System.

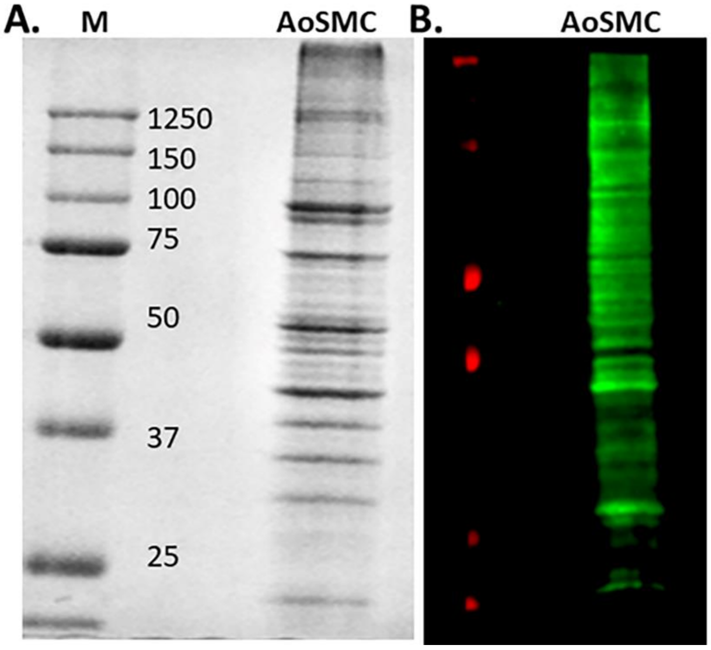

Figure S2. Coumassie Stain (A) and Western blot (B) of biotin-labelled AoSMC preparation used as bait in the pull-down assay.

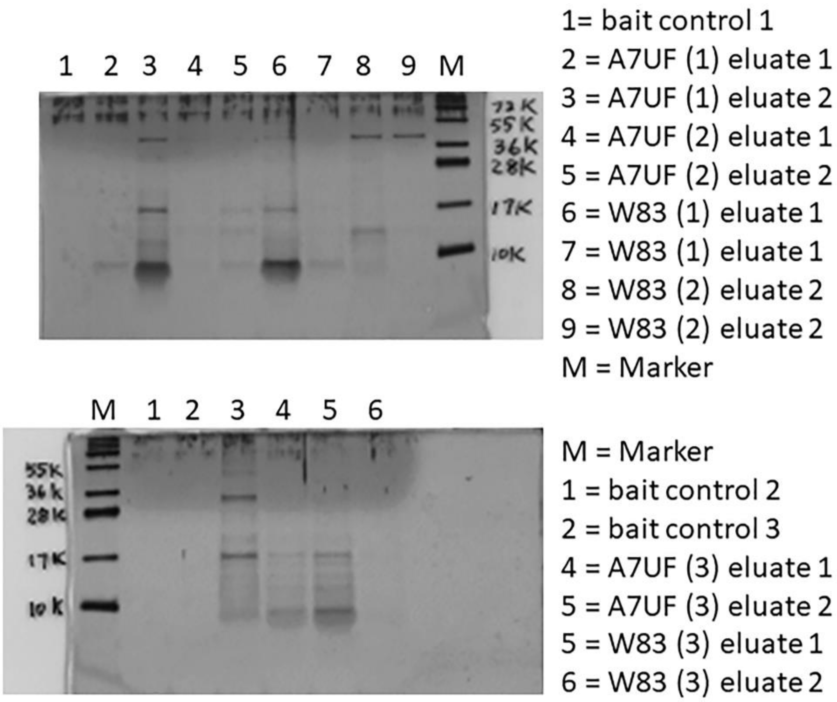

Figure. S3 Silver stained gel of serial pull-down optimization experiments.

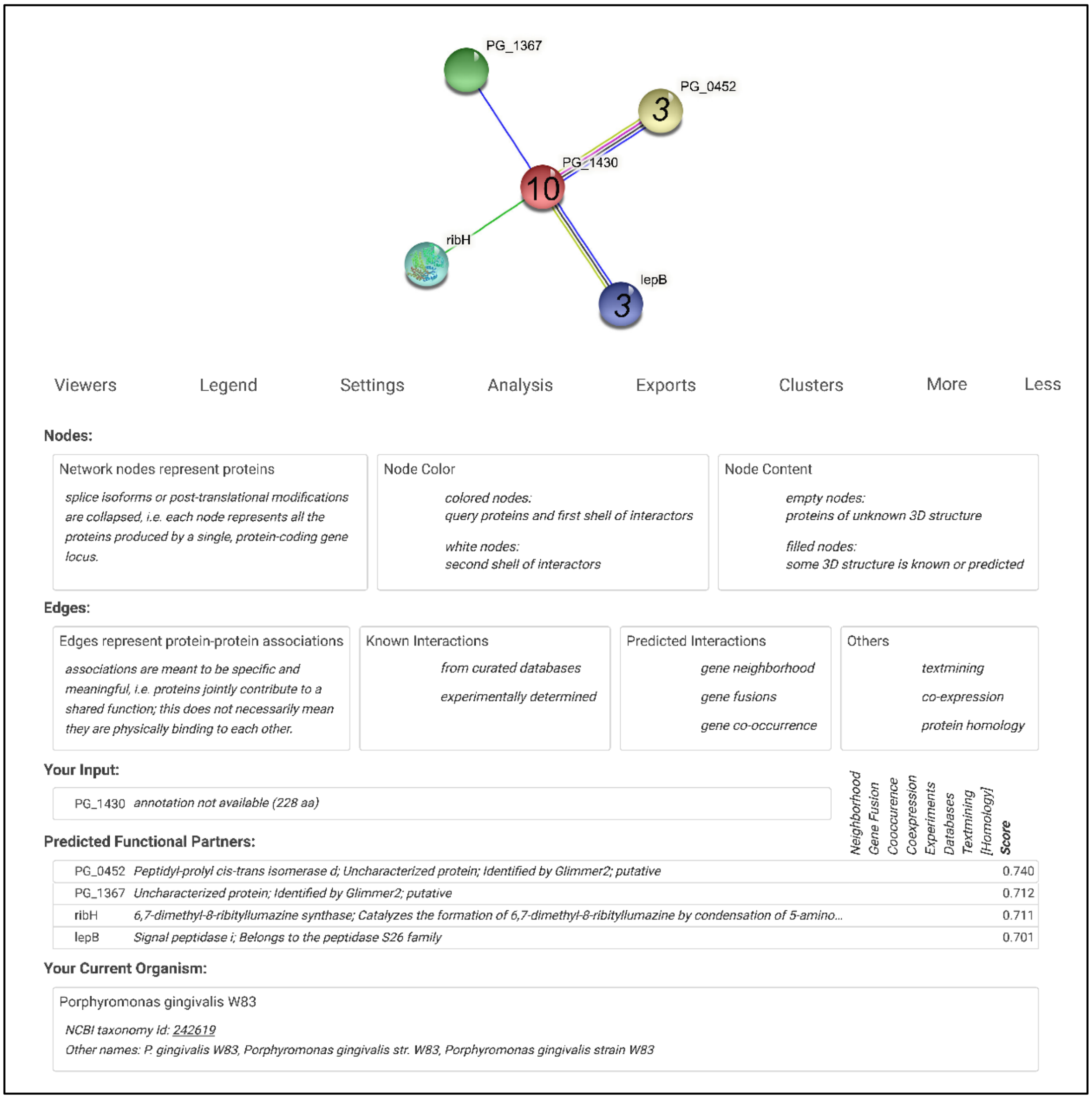

Figure S4. PG_143 interaction network using STRING database.org. Regular text in nodes indicate the spectral counts of the protein found in the A7UF eluate. Italicized numbers within nodes indicate the fold greater number of spectral counts in the A7UF eluate compared to W83. Nodes without numbers indicate proteins that were not identified in the pull-down eluates.

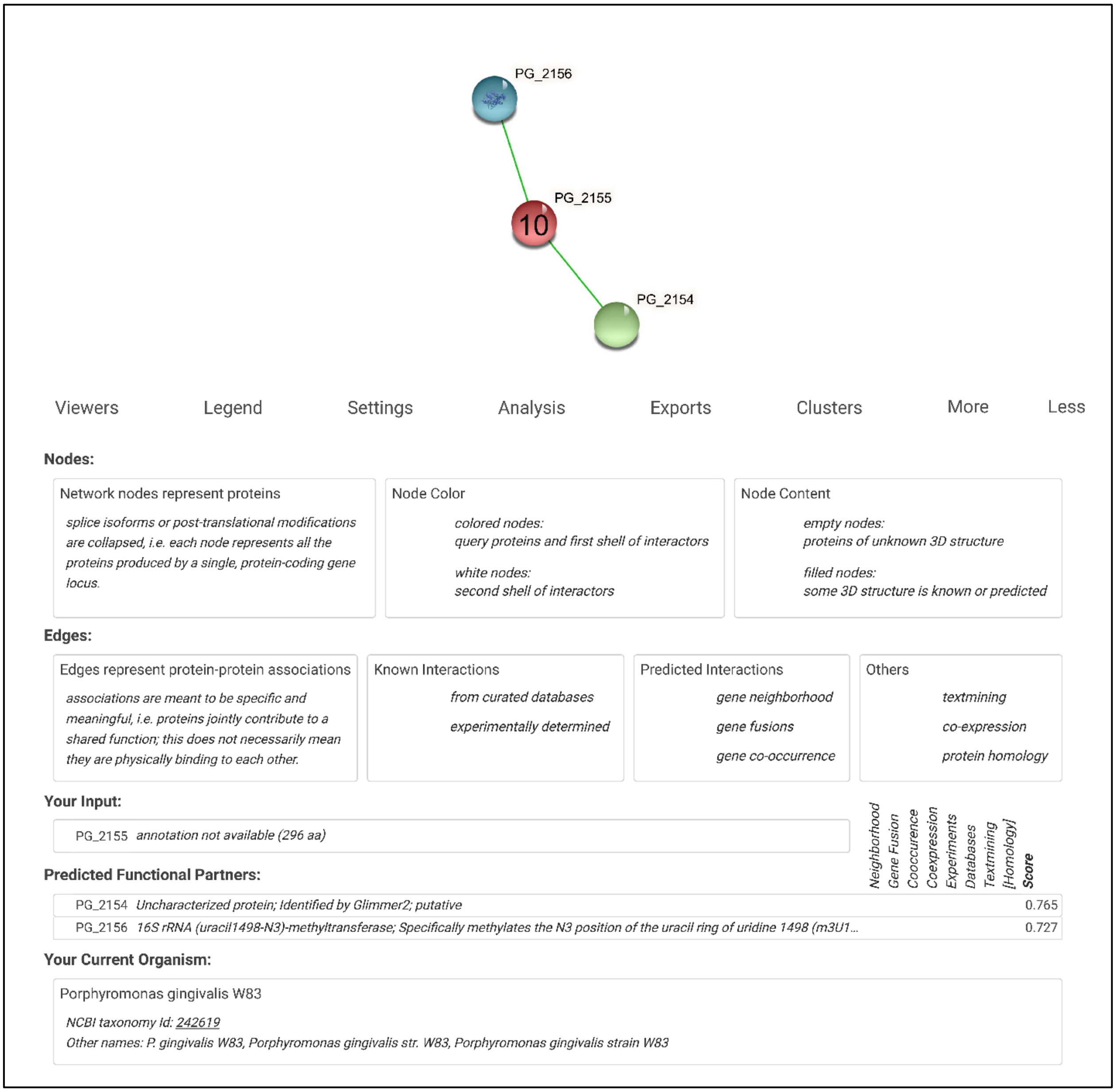

Figure S5. PG2155 interaction network using STRING database.org. Regular text in nodes indicate the spectral counts of the protein found in the A7UF eluate. Nodes without numbers indicate proteins that were not identified in the pull-down eluates.
